# Assessment of Lead (Pb) Leakage From Abandoned Mine Tailing Ponds to Klity Creek, Kanchanaburi Province, Thailand

**DOI:** 10.1029/2020GH000252

**Published:** 2021-05-01

**Authors:** Supawan Srirattana, Kitsanateen Piaowan, Thanyathit Imthieang, Jiraporn Suk ‐in, Tanapon Phenrat

**Affiliations:** ^1^ Department of Civil Engineering Faculty of Engineering Naresuan University Phitsanulok Thailand; ^2^ Research Unit for Integrated Natural Resources Remediation and Reclamation (IN3R) Department of Civil Engineering Faculty of Engineering Naresuan University Phitsanulok Thailand; ^3^ Center of Excellence for Sustainability of Health Environment and Industry (SHEI) Faculty of Engineering Naresuan University Phitsanulok Thailand

**Keywords:** abandoned tailing ponds, groundwater and sediment contamination, Pb radionuclide, seepage, stable Pb isotope ratios

## Abstract

In 2013, Klity Creek became the site of Thailand's first legally required remediation, 15 years after the spill of lead (Pb)‐contaminated mine tailings into the creek. Even today, nature cannot attenuate Pb‐contaminated sediment, arguably due to either high geological background Pb or continuous leakage of Pb from the unlined tailing ponds, upstream of the creek. In this study, four lines of evidence were used to reveal that the leakage from tailing ponds is primarily responsible for the long‐term Pb contamination. First, stable Pb isotope ratios (^206^Pb/^207^Pb and ^208^Pb/^207^Pb) were used to apportion sources between the tailings and geological background. The analysis of samples from the tailing ponds, geological background, and local zinc (Zn)‐Pb deposit revealed five different Pb sources (i.e., two distinct mine tailings, two different backgrounds, and a local Zn‐Pb deposit) in the area based on five unique isotope ratios. Using source apportionment analysis, Pb‐contaminated sediments in Klity Creek were consistent with tailings being the dominant source (30%–100%). Likewise, an analysis of Pb radionuclide (^210^Pb) revealed the Pb in the contaminated sediment was relatively new, 0–6.7 years old, suggesting that the Pb source was recent leakage from the tailing ponds rather than the 15‐year‐old tailing spill. Isotope evidence was supported by the elevated Pb‐contaminated seepage (0.30 ± 0.22 mg/L) from the tailing ponds and groundwater samples (up to 0.225 mg/L) collected from monitoring wells surrounding the tailing ponds. Consequently, proper management of Pb leakage from the tailing ponds is critical for successful Klity Creek remediation.

## Key Points

- Resolution of a 20‐year debate on the source of Pb pollution in Klity Creek
- Mine tailings are still leaking from abandoned tailing storage ponds
- The source of Pb in Klity Creek is predominantly mine tailing and Kilty Creek remediation requires elimination of mine tailing leaks

## 1 Introduction

Klity Creek in Kanchanaburi's Thong Pha Phum district is the most infamous Pb mining site in Thailand, attracting significant international attention (Pearshouse, 2014; Phenrat et al., 2016). In 1998, ∼17,540 metric tons of Pb‐contaminated mine tailings spilled from tailing ponds into the creek and surrounding areas, causing severe Pb contamination (Panichayapichet et al., 2008; Pedall et al., 1999). Because Klity Creek is the only water source for more than 400 villagers (Phenrat et al., 2016), providing water for not only consumption but also aquaculture and agriculture, exposure of the local inhabitants was unavoidable (Pusapukdepob et al., 2007).

More sensitive to Pb poisoning than adults, children with blood lead levels (BLLs) greater than 10 μg/dL are at higher risk for developmental disabilities; each μg/dL BLL increase between 5 and 35 μg/dL has been reported to correspond to an IQ decrease of 2–4 points (H.‐B. Li et al., 2015; Pusapukdepob et al., 2007). Phenrat et al. (2016) estimated the BLL of children living in Klity Creek villages using the integrated exposure uptake biokinetic model and found that up to 99.26% have a BLL greater than 10 µg/dL. Pusapukdepob et al. (2007) reported that the Klity villagers, especially children, tended to score lower on IQ tests and more frequently reported nausea, vomiting, abdominal pain, constipation, muscle pains, headaches, insomnia, and memory loss, all common symptoms for Pb poisoning (Pusapukdepob et al., 2007).

Sued by the affected villagers since 2004, the Pollution Control Department (PCD, a government agency) was ordered by the court in 2013 to restore Klity Creek so that Pb in and around the entire contaminated environment—water, sediment, aquatic animals, soil, and plants—must fall below a certain level on four sampling events over one year (Phenrat et al., 2016). The budget for the first phase of this restoration project is USD 15.25 million, and all stakeholders agreed that the remediation will not be easily achieved. In 2016, more than 18 years after the spill, the Pb concentrations in the affected sediment were still extremely high (up to 160,000 mg/kg). Some stakeholders and Phenrat et al. (2016) contended that Pb is still leaking from tailing ponds to the soil and Klity Creek, and although natural recovery of metal‐contaminated sediment is possible elsewhere (Fuchsman et al., 2014; Moore & Langner, 2012), Klity Creek has no substantial natural recovery potential for sediment except at the station (KC8) furthest from the tailing ponds (Phenrat et al., 2016).

Although continuous Pb leakage from the abandoned and unlined tailing ponds could be partially responsible for the poor natural recovery potential, some stakeholders attribute the long‐term contamination to high geological background Pb in the area that continuously adds Pb to the creek by soil erosion (Thaipublica, 2014). Certainty regarding the source of Pb is critical because if natural geology is the major contributor of Pb, the restoration will be impossible. Alternatively, if the major contribution of Pb in Klity Creek sediment is from the tailing ponds, it would be critical to prevent their further Pb leakage. In this study, we utilized four lines of evidence, including stable Pb isotope from mine tailings and the geological background, a Pb radionuclide of Klity Creek's sediment, and Pb concentration in seepage and in groundwater samples from the tailing ponds to determine the source of Pb contamination in the sediment of Klity Creek.

Lead is present in the environment as four main isotopes: ^208^Pb (52%), ^206^Pb (24%), ^207^Pb (23%), and ^204^Pb (1%), while radiogenic isotopes ^206^Pb, ^207^Pb, and ^208^Pb are products of the radioactive decay of ^238^U, ^235^U. and ^232^Th, respectively. ^204^Pb is the only primordial stable isotope with a constant abundance on Earth over time (Komárek et al., 2008). The isotopic composition of Pb is commonly expressed as ratios ^206^Pb/^204^Pb, ^208^Pb/^207^Pb, ^208^Pb/^206^Pb, and ^206^Pb/^207^Pb, the last of which is generally preferred because it can be precisely measured and the abundances of these particular isotopes are considered relatively important (Komárek et al., 2008). Furthermore, the global abundance of ^207^Pb is very stable because most ^235^U has already decayed, while ^238^U is still relatively abundant (Erel et al., 2001). Relatively old Pb ores are generally characterized by a low ^206^Pb/^207^Pb ratio (1.06–1.10), and more recent samples containing more radiogenic Pb originating from U and Th decay demonstrate higher ^206^Pb/^207^Pb ratios (>1.18) (Bacon, 2002; Farmer et al., 2000). Stable Pb isotopes can be effective tools for environmental forensics, as reported in several cases cited in the supporting information (SI) (Figure S1 and Table S1), which address issues similar to those investigated in this study (Akande & Zentilli, 1983; Arribas & Tosdal, 1994; Bjorlykke et al., 1993; Brigo et al., 1977; Cheng & Hu, 2010; Coron, 1981; Cumming et al., 1979, 1990; Cumming & Richards, 1975; Deb et al., 1989; Gemmel et al., 1992; Godwin et al., 1988; Godwin & Sinclair, 1982; Gulson, 1984, 1985, 1986; Heal, 1976; Hou & Zhao, 1993; Höy & Godwin, 1988; James & Henry, 1993; Koeppel, 1978; Koppel & Schroll, 1983, 1988; Lang & Zhang, 1987; Lange et al., 1985; LeCouteur & Clifford, 1973; Léon, 1998; N. Li, 1998; Macfarlane et al., 1990; McCracken, 1997; Morganti, 1979; Morrow & Cumming, 1982; Puig, 1990; Rui et al., 1991; Sangster et al., 1998; Sangster & Vaillancourt, 1990; Sato & Sasaki, 1973; Shanks et al., 1987; Song et al., 1996; Tompkins et al., 1994; Tornos & Arias, 1993; Vaasjoki & Gulson, 1986; Vearncombe et al., 1995; Velasco et al., 1996; Walker et al., 1983; Zartman et al., 1979).

On the other hand, natural ^210^Pb radionuclide profiles of bottom sediment core samples are commonly used for dating river sediment to trace the history and sources of pollution (Gelen et al., 2003; Shotyk et al., 1996; Vile et al., 2000). In a sediment sample, there are two types of radioactive ^210^Pb. The first is excess ^210^Pb, which is the solid fallout of a decay product of gaseous ^222^Rn initially released to the atmosphere from the soil surface, which will be deposited onto the surface soil or water reservoirs within a couple of weeks. This ^210^Pb will be adsorbed onto each layer of surface sediment, generating excess ^210^Pb activity on top of the second type of ^210^Pb produced inside the sediment matrix (Lubis, 2006), which is considered under radioactive equilibrium with ^226^Ra, its long‐lived precursor. Consequently, excess ^210^Pb can be easily determined from the total ^210^Pb and the ^210^Pb produced inside the sediment matrix.

The excess ^210^Pb can be used to determine the sediment age because the excess ^210^Pb decays with age according to the radioactive decay law (half‐life [T_1/2_] = 22.3 years) (Lubis, 2006). Thus, by assuming that (1) every sediment layer has the same initial excess ^210^Pb activity, (2) fallout of ^210^Pb from the atmosphere to the water is constant, resulting in a constant rate of ^210^Pb deposit to the sediments irrespective of any variation, and (3) excess ^210^Pb activity will decline exponentially with increasing depth of sediment, the constant rate of supply (CRS) model can be used to determine the sediment age and accumulation rates which vary with depth and over time (Crickmore et al., 1990; Ivanovich & Harmon, 1992; Lubis, 2006). The CRS model is most widely used for aquatic systems, where sedimentation processes are intensified by anthropogenic activities (Lubis, 2006). The uncertainty of sediment dating using the excess ^210^Pb decays at 95% confidence intervals is 1–2 years at 10 years of age, 10–20 at 100 years of age, and 8–90 at 150 years of age (Binford, 1990).

The aim of this study was to systematically assess if the leakage from tailing ponds is primarily responsible for the long‐term Pb contamination in Klity Creek. This assessment utilized four lines of evidence. First, stable Pb isotope ratios (^206^Pb/^207^Pb and ^208^Pb/^207^Pb) were used to apportion sources between the tailing and geological background. Second, the analysis of ^210^Pb was used to investigate if the Pb in the contaminated sediment is relatively new, Pb that recently leaked from the tailing storage ponds, or relatively old, from the 15‐year‐old tailing spill. Finally, this study also analyzed Pb concentration in observed seepage samples as well as groundwater samples from the monitoring wells surrounding the tailing ponds to support the findings from Pb isotope analysis. The present study is the first to systematically investigate Pb leakage from the tailing ponds to Klity Creek. These results may assist in guiding cost‐effective creek restoration strategies to meet remediation goals ordered by the court.

## 2 Methodology

### 2.1 Study Area

**Figure 1:**
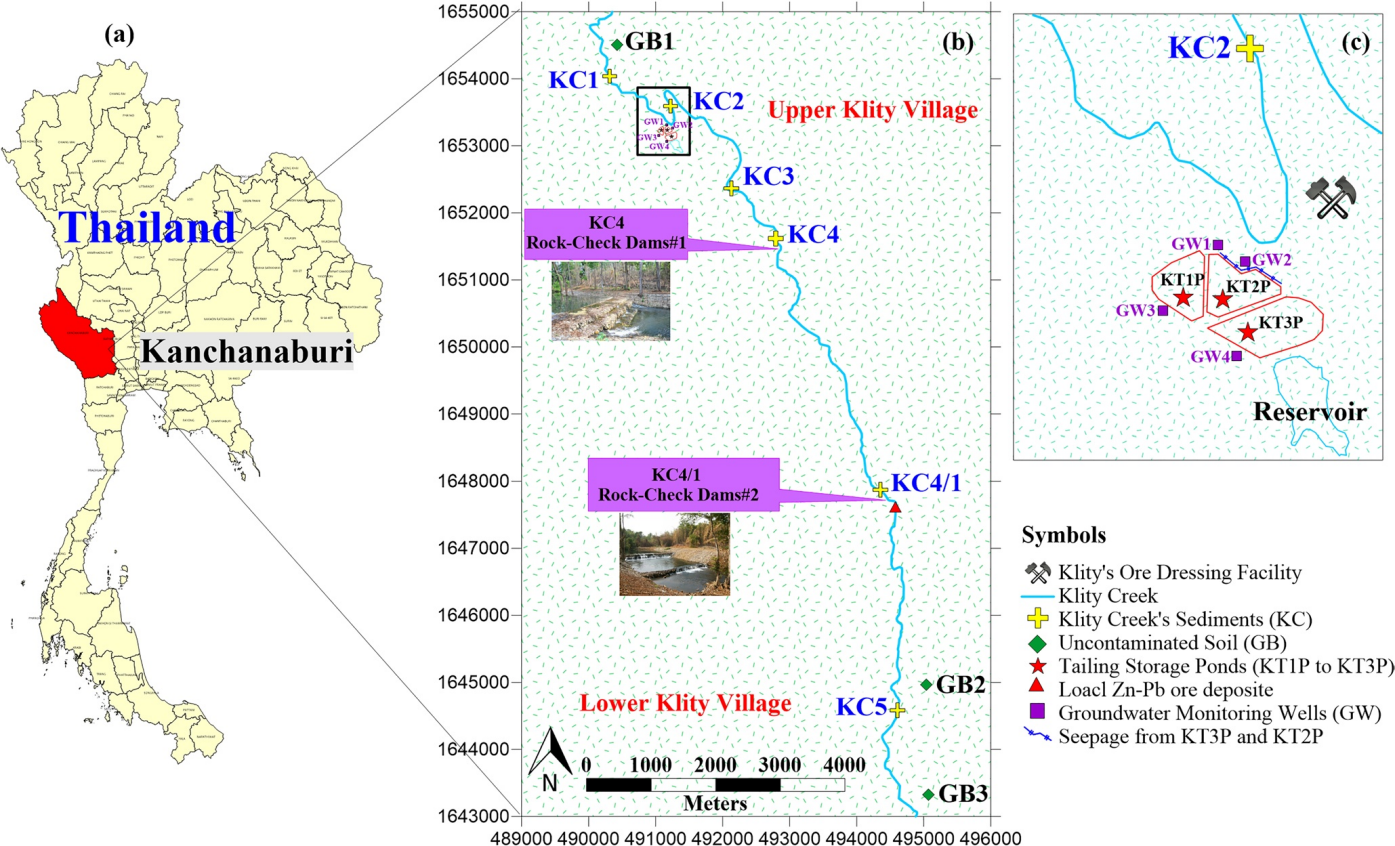
(a) A map of Kanchanaburi province, Thailand (red), and a map of Klity Creek (b–c) with the three tailing storage ponds (KT1P to KT3P), six locations of composite Klity Creek's bottom sediment stations (KC1 to KC5), seepage location, and four groundwater monitoring wells (GW1 to GW4).

The portion of Klity Creek assessed in this study (Figure 1) is from KC1 to KC5 (10.5 km along the creek), the portion most heavily contaminated (Phenrat et al., 2016). Notably, KC1 to KC5 are the official water and sediment quality monitoring sites which have been sampled to track the change of Pb concentration in the creek by PCD for more than 20 years (i.e., since the spill to the present) (Phenrat et al., 2016). Two affected communities living along this part of the creek include the Upper Kilty Village located close to KC2 and the Lower Klity Village located close to KC5. KC1 is upstream of the ore dressing facility, while KC2 to KC5 are downstream of the spill point. KC2 is where the spill occurred while KC4/1 and KC4 are near two rock‐check dams constructed by the mine to block the migration of the Pb‐contaminated sediment from the spill. Moreover, KC4/1 is also close to the vicinity of the local Zn‐Pb ore deposit of the area.

Three tailing storage ponds, KT1P, KT2P, and KT3P, are located close to KC2 in the Upper Klity Village (Figure 1). Of the three tailing ponds, KT1P is never used (empty), whereas KT3P is the largest with an area of 14,144 m^2^ and a depth of 6 m. It stores the largest mass of Pb mine tailings (684,625 tons) (PCD, 2010, 2014). Similarly, KT2P has an area of 8,864 m^2^ and a depth of 6 m, containing 204,483 tons of Pb mine tailings. Upstream of the KT2P and KT3P is a full natural water reservoir (Figure 1). The mine tailings are residual from dressing Pb crude ore of the Bo Ngam mine (∼10 km from the facility) and consist of galena (PbS), cerussite (PbCO_3_), and some anglesite (PbSO_4_) in limestone, clay, and quartz matrices with average Pb concentrations of 3%–4% (30,000–40,000 mg/kg) by weight (Phenrat et al., 2016). The mine tailing have been classified as hazardous waste as the Pb concentration in the leachate was determined to be 4,056 mg/L (PCD, 2010) using methods equivalent to the U.S. EPA Method 1311, toxicity characteristic leaching procedure (U.S. EPA, 1992). The tailing ponds have neither clay, HDPE liner, nor monitoring wells (Figure 1).

### 2.2 Timeline of Four Lines of Evidence

**Figure 2:**
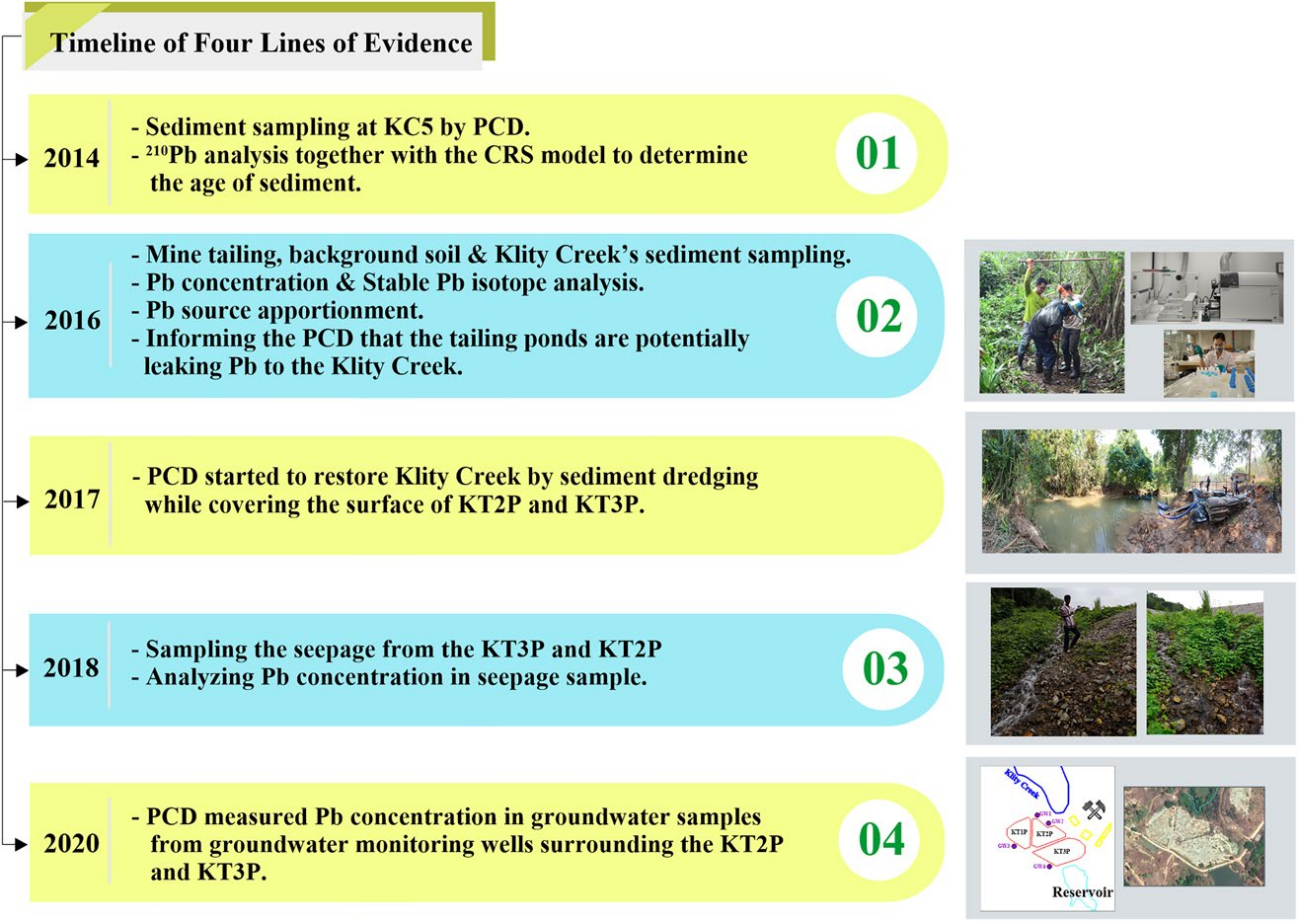
Timeline of four lines of evidence.

Samples for Pb contamination were collected during four collection events (Figure 2). To determine the age of Klity Creek's sediment at different depths, we utilized the PCD findings from 2014, for which a total of 34 sediment core samples were collected at site KC5 over a depth of 1.4 m. The PCD used the natural radionuclide ^210^Pb, together with the CRS model, to determine the age of KC5 sediment at multiple depths (PCD, 2014). The second sampling event occurred in 2016. We collected samples from KT2P and KT3P to assess Pb contamination and isotope characteristics of mine tailings, and from noncontaminated soil samples at high elevation in both the Upper and the Lower Klity areas to measure geological background Pb. The local Zn‐Pb ore deposit close to KC5 was also analyzed as a potential natural, local source of Pb. After obtaining the isotope results from the first and second field sampling events, we informed the PCD that the tailing ponds are potentially leaking Pb to the Klity Creek. Nevertheless, no remedial action other than covering the surface of the tailings pond was performed by PCD. In 2018, we analyzed Pb from samples of the seepage along the edge of the KT3P and KT2P (Figure 1). In 2020, we obtained the Pb concentrations in groundwater monitoring wells surrounding the KT2P and KT3P, as measured by PCD. We assessed these four lines of evidence to conclude whether the mine tailings Pb is still entering the creek. The details of each sampling event are discussed below.

### 2.3 Mine Tailing, Local Zn‐Pb Deposit, Uncontaminated Geological Background Soil, and Sediment Sampling in 2016

#### 2.3.1 Location and Sampling Protocol

The four types of samples collected for the second sampling event are summarized in Table 1, together with their descriptions and locations. Sampling location details are shown in Figure 1. The first type is the mine tailing samples. A total of 7 and 10 mine tailing samples were collected from inside the KT2P and KT3P at depths of 0–175 cm and 0–300 cm below the ground, respectively, using a hand auger. We focused our investigation at these depths because, in 2010, the PCD performed electrical resistivity tomography and took core samples of KT2P and KT3P, finding high Pb concentrations at ∼175 cm and 300 m from the surface at KT2P and KT3P, respectively (PCD, 2010).

**Table 1:** Summary of Mine Tailings, Uncontaminated Geological Backgrounds, Local Zn‐Pb Source, and Klity Creek's Sediment Sampled in 2016

No.	Latitude	Longitude	ID	Depth (m)	No. of samples	Description
Mine tailing samples							
1	14.954020°	98.918181°	KT2P	1.75	7	Mine tailing from KT2P	
2	14.953414°	98.918842°	KT3P	3	10	Mine tailing from KT3P	
Composite geological background samples							
3	14.973528°	98.910917°	GB1	0.75	1	Uncontaminated soil in Upper Klity Village	
4	14.878083°	98.952000°	GB2	0.75	1	Uncontaminated soil in Lower Klity Village	
5	14.864277°	98.953549°	GB3	0.75	1	Uncontaminated soil in Lower Klity Village	
Composite local Pb Source							
6	14.903904°	98.948380°	LS1	–	1	Local Zn‐Pb ore close to KC4/1	
Composite Klity Creek's bottom sediment							
7	14.961780°	98.909790°	KC1	0.3	1	Uncontaminated sediment from KC1 in Upper Klity Village	
8	14.957718°	98.918468°	KC2	0.3	1	Contaminated sediment at the spill point in the Upper Klity Village	
9	14.953470°	98.924775°	KC3	0.3	1	Contaminated sediment at KC3 in Upper Klity Village	
10	14.942803°	98.930163°	KC4	0.3	1	Contaminated sediment at KC4, rock‐check dam midway between Upper and Lower Klity Village	
11	14.904771°	98.947106°	KC4/1	0.3	1	Contaminated sediment at KC4/1, a rock check dam midway between Upper and Lower Klity Village	
12	14.877657°	98.949511°	KC5	0.3	1	Contaminated sediment at KC5 at Lower Klity Village	

The second type of samples was the uncontaminated geological background. We obtained composite soil samples from three uncontaminated areas in the Upper‐ and the Lower Klity Villages. GB1 was from the Upper Klity Village while GB2 and GB3 were from the Lower Klity Village. These locations are at a higher elevation than Klity Creek and its flood plain and thus not affected by spilled tailing waste. As a result, these samples serve as the unimpacted geological background. For each composite sample, four random soil samples at each location were collected using a hand auger from 0 to 75 cm depth. The third type of sample was the local Pb source. This sample is the LS1 collected from the local Zn‐Pb deposit near KC4/1 and represents potentially high Pb contamination to the Klity Creek from the local, natural source.

The last type of sample is the Klity Creek's bottom sediment. Composite bottom sediment samples were collected from six locations (KC1, 2, 3, 4, 4/1, and 5) at a depth of 0–20 cm, using a 7‐cm diameter coring tube. For each location, four random bottom sediment samples were collected to make a composite sample. These samples were analyzed for Pb source apportionment from the mine tailings, the geological background, and the local Pb source using stable Pb isotope analysis. Notably, only sediment samples were collected from Klity Creek because 20 years after the spill, it is the sediment (and not the water) that remains heavily contaminated and is the component considered in the debate of the origin of high Pb contamination, as described in the introduction. We named the sediment samples according to their location, KC1 to KC5, the official water and sediment quality monitoring sites, for consistency. However, sediment samples KC1 to KC5 in this study are not identical to the KC1 to KC5 samples reported in Phenrat et al., 2016. They were collected from the same sampling station but at different sampling times (in 2013–2014 for Phenrat et al., 2016 vs. 2016 for this study).

#### 2.3.2 Sample Preparation and Digestion

First, soil and sediment samples were dried in a hot‐air oven at 80°C to constant weight. Second, the samples were sieved through a 20‐mesh screen (0.841 mm) (Phenrat et al., 2016) prior to digestion based on the U.S. EPA Method 3051a (U.S. EPA, 2007). Duplicates were performed for each sample. A microwave‐assisted acid digester (ETHOS EASY, Milestone, Italy) was used for acid digestion of the samples. In this method, 0.25 g of sample was weighed and mixed with 10 mL 70% HNO_3_ in a PTFE reactor. The reactor was sealed and heated following a two‐stage microwave digestion program. The first stage required 10 min to reach 180°C, and the second stage, 10 min at 180°C. After cooling, sample digests were passed through a Whatman 42 filter, transferred into a 25 mL flask, and brought to volume with HNO_3_ (1%). All the reagents were analytical grade.

#### 2.3.3 Pb Solution Analysis

The digested samples were used to analyze Pb concentrations and isotope ratios (^206^Pb/^207^Pb and ^208^Pb/^207^Pb). At the Thailand Institute of Nuclear Technology laboratory (TINT), an accredited national laboratory for both Pb stable isotope and ^210^Pb, the samples were analyzed by ICP‐MS (Agilent 7900 with Agilent ICP‐MS MassHunter Software (Figure S2 in SI), Agilent, Santa Clara, US) to obtain trace metal concentrations and isotopic compositions. ICP‐MS instrument operating conditions are shown in Table S2 in SI. Appropriate QA/QC measures were performed every day as standard procedure at the TINT. In every working day, a standard calibration curve (0, 5, 10, 25, and 50 ppb) of Pb solution (NIST SRM 981) was analyzed to determine the sensitivity (Figure S3 and Table S3 in SI). After measuring the Pb concentration in digested samples, the solutions were adjusted to a Pb concentration of ∼30 μg/L, according to the suitable working range (5–50 μg/L) selected previously. Dilutions were spiked with a 209‐Bi high purity solution to 1 μg/L to correct for mass bias drift (Figure S4 in SI). Moreover, the measurement procedure was evaluated by analyzing a 25 and 50 μg/L NIST SRM 981 solution at the beginning and after every five samples to check the instrumental drift.

#### 2.3.4 Source Apportionment Analyses

We assumed that the Pb in the geological background samples would have specific Pb isotopic characteristics and concentrations different from that in the mine tailings, while the other Pb‐contaminated sediment samples in Klity Creek would be a mixture of Pb from these two sources (Yu et al., 2016). Thus, source apportionment analyses were performed to determine the contribution of mine tailings and natural geological background to Pb concentrations in sediment samples in Klity Creek (KC1 to KC5).

We first ascertained the isotope characteristics of mine‐tailing samples from the isotope ratio (^206^Pb/^207^Pb and ^208^Pb/^207^Pb) of mine tailing samples obtained from KT2P and KT3P, which were confirmed by their characteristic appearance (i.e., unnatural fine black particles, as also described by PCD (PCD, 2014). We obtained the isotope characteristics of the natural geological background from the same isotope ratio of the GB1, GB2, and GB3 samples. Moreover, the isotope characteristics of the local Pb source was also determined for the same isotope ratio of the local Zn‐Pb ore sample.

Consequently, from these analyses, we obtained the isotopic and concentration characteristics of the mine tailing sources, geological background, and local source. Next, we applied trial‐and‐error analysis modified from Yu et al. (2016) to determine the most probable contribution of Pb from the different sources that satisfied the isotopic and total concentration of Pb in the other sediment samples (Yu et al., 2016). The sources were assumed to contribute 100% of the Pb in each sediment sample. The four trial‐and‐error parameters were (206/207)_j_, (208/207)_j_, (Pb concentration)_j,_ and *k*_j,i_, which represent the trial ^206^Pb/^207^Pb ratio, ^208^Pb/^207^Pb ratio, Pb concentration, and mass proportion of end members j, respectively, for sample i.

As detailed in the results and discussion, the analyses of isotope characteristics yielded five distinct sources of Pb in the Klity area, including two distinct mine tailing sources (TS1 and TS2) from the tailing ponds, a local Zn‐Pb ore (LS1), two distinct geological backgrounds from Upper Klity area (BS1), and geological background from Lower Klity area (BS2). As a result, the j represented the five end members with *j* = 1 for TS1, *j* = 2 for TS2, *j* = 3 for LS1, *j* = 4 for BS1, and *j* = 5 for BS2. The trial‐and‐error process sampled (206/207)_j_, (208/207)_j_, and (Pb concentration)_j_ within the range of ^206^Pb/^207^Pb, ^208^Pb/^207^Pb, and Pb‐concentration values from those characteristic values that represent the geological background and mine tailing for *j* = 1 to 5 obtained in the previous step. It sampled *k*_j,i_ from 0 to 1. The sum of all *k*_j,i_ in each simulation must be equal to 1. Then, for each trial‐and‐error condition, we calculated (^206^Pb/^207^Pb)_c,i_, (^208^Pb/^207^Pb)_c,i_, and (Pb concentration)_c,i_ representing the calculated ^206^Pb/^207^Pb ratio, ^208^Pb/^207^Pb ratio, and Pb concentration, respectively, for sample i at trial *k*_j,i_ (see Equations (1), (2), (3), (4)).

(1) $${\left(208/207\right)}_{c,i}=\overset{5}{\underset{j=1}{\sum }}k_{j,i}{\left(208/207\right)}_{j}$$

(2) $${\left(206/207\right)}_{c,i}=\overset{5}{\underset{j=1}{\sum }}k_{j,i}{\left(206/207\right)}_{j}$$

(3) $$\left(\text{Pb}\;\text{concentration}\right)c,i\;=\overset{5}{\underset{j=1}{\sum }}k_{j,i}{\left(\text{Pb}\;\text{concentration}\right)}_{j}$$

(4) $$\overset{5}{\underset{j=1}{\sum }}k_{j,i}=1,\;\text{where}\;0\leq k_{j,i}\leq 1$$

Next, we compared the calculated ^206^Pb/^207^Pb ratio, ^208^Pb/^207^Pb ratio, and Pb concentration for sample i with the measured values ((208/207)_i_, (206/207)_i_, and (Pb concentration)_i_) from the field sample i. A least‐square optimization method was used to minimize the sum‐of‐square differences between the calculated and observed isotope ratios and Pb‐concentration values (Equation 5):

(5) $$\begin{array}{l} F\left(k_{j,i}\right)\;=\;min\sum (\;{\left({\left(208/207\right)}_{c,i}-\;\left(208/207\right)i\right)}^{2}\\ \;+\;{\left({\left(206/207\right)}_{c,i}-\;{\left(206/207\right)}_{i}\right)}^{2}\\ +\;{\left({\left(\text{Pb}\;\text{concentration}\right)}_{c,i}-\;{\left(\text{Pb}\;\text{concentration}\right)}_{i}\right)}^{2}) \end{array}$$

The most probable source apportionment is the trial ^206^Pb/^207^Pb ratio, ^208^Pb/^207^Pb ratio, Pb concentration, and *k*_j,i_, which yielded the smallest F (*k*_j,i_) value. MATLAB (2017a) was used in this trial‐and‐error process. Lsqlin in MATLAB R2017a was used to obtain the optimized solution; the mass proportions of different end members for each sample were calculated, and results were shown as a source percentage of each sample.

### 2.4 Age of Sediment Analyses

In 2014, PCD collected a total of 34 sediment core samples from KC5 at a depth of 0–1.4 m. Samples were taken at every 0.02 m for the first 0.5 m deep and at every 0.1 m for 0.5–1.4 m deep. Sediment from each depth interval was transferred into precleaned and pre‐weighed polyethylene vials for subsequent determination of radionuclide concentrations (Srisuksawad et al., 1997). The radionuclide ^210^Pb (via detection of its granddaughter, ^210^Po), which can be measured in various media, including water, rocks, soil, and sediment (Ebaid & Khater, 2006), was analyzed using alpha‐particle spectrometry by an accredited laboratory at TINT. These results were published in PCD (2014).

A summary of the TINT standard protocol for ^210^Pb quantification via ^210^Po measurement is as follows. First, the samples were oven‐dried to constant weight (less than 24 h) at 60°C prior to being crushed, ground, and sieved through a 20‐mesh screen (0.841 mm) to fine homogeneous powders. Next, for quality assurance, 2–3 g of ^209^Po yield tracer (provided by the International Atomic Energy Agency) was added as an internal standard to each dried sample before microwave‐assisted acid digestion. The pressure, microwave power, temperature, and time were optimized for digestion efficiency. Centrifugation was used to separate digested residual solids from the solution. Ascorbic acid was added to the solutions immediately before they were deposited onto silver discs to prevent Fe deposition. Finally, alpha‐particle emissions of ^209^Po (4,887 keV) and ^210^Po (5,305 keV) from these discs were measured for 24–48 h by alpha spectrometry. The chemical yield of ^210^Po deposition (in units of disintegrations per minute [dpm] per gram of sample), which is equivalent to ^210^Pb, was calculated using Equation 6 (Carpenter et al., 1981; Chanyotha & Kritsananuwat, 2018; Srisuksawad et al., 1997):

(6) P210o=P209o×peakarea210Popeakarea209Po×sampleweight

Next, the CRS model, proposed by Krishnaswamy et al. (1971), was employed in this analysis. The model illustrates the relationship between the cumulative residual excess ^210^Pb (A) beneath sediments of age t and the total residual excess ^210^Pb in the sediment column (A(o)) as well as the ^210^Pb radioactive decay constant (*k* = ln (2)/T_1/2_) in Equation 7 (Lubis, 2006):

(7) $$A\;=\;A\left(o\right)e{-}^{kt}$$

A and A(o) are calculated by direct numerical integration of the ^210^Pb profile. The age of sediments of a particular depth of interest is then given in Equation 8 (Lubis, 2006):

(8) $$t=\frac{1}{k}\ln\frac{A\left(o\right)}{A}$$

The sediment accumulation rates can be calculated from Equation 9 where C is the ^210^Pb concentration in the sediment:

(9) $$r=\frac{\mathrm{kA}}{C}$$

### 2.5 Seepage Samples From KT3P and KT2P in 2018

In 2018, while PCD's remediation contractor was filling the KT2P and KT3P with crushed rock to prepare for surface covering of the tailing ponds by soil and vetiver grass, a large amount of seepage at the embankment of the tailing ponds was observed (Figure 2). Three samples from three locations of the seepage were collected, preserved, and analyzed for the total Pb concentration by ICP‐MS (Method 3125, APHA) at an accredited laboratory service.

### 2.6 Pb Concentration in Groundwater Samples Surrounding the Tailing Ponds in 2020

PCD installed a total of four groundwater monitoring wells (GW1 to GW4 in Figure 1) surrounding the tailing ponds in 2019 and collected groundwater samples to analyze both dissolved and total Pb in 2020 using an accredited laboratory service by ICP‐MS (Method 3125, APHA). GW3 is used to monitor upgradient of KT1P, and GW4 is used to monitor upgradient of KT3P, while GW1 and GW2 are used to monitor the downgradient of KT2P and KT3P. The data was presented in a triparty committee meeting and an author of this article (T. Phenrat) served on the committee. We then used this data as the last and most up‐to‐date line of evidence to combine with the other two, Pb source apportionment and age of sediment.

## 3 Results and Discussion

### 3.1 Pb Content in Mine Tailings, Local Zn‐Pb Deposit, Uncontaminated Geological Background Soil, and Sediment Sampling in 2016

The total Pb concentrations of all mine tailings, the local Zn‐Pb deposit, and the uncontaminated geological background soil samples obtained in 2016 are shown in Table S4. Several findings for each group of samples are summarized here.

#### 3.1.1 Pb Content in Uncontaminated Soil Samples and Local Zn‐Pb Deposit

**Figure 3:**
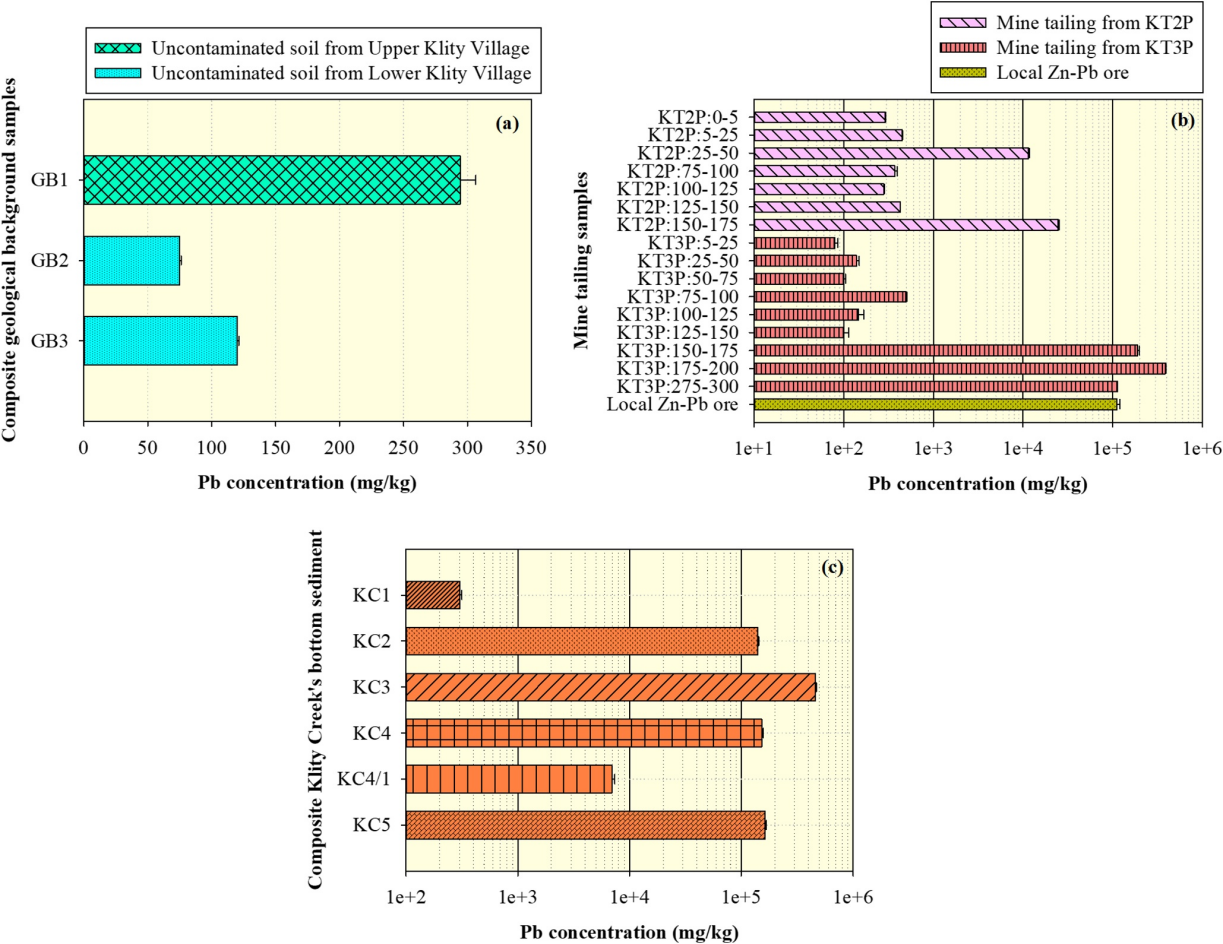
(a) Lead content in uncontaminated soil samples, (b) lead content in samples from tailings storage ponds (codified as location: depth (m)) and local Zn‐Pb deposit, and (c) lead content in sediment samples.

The Pb concentration in soil collected from three uncontaminated areas were 294.40 ± 12.19 (GB1), 74.74 ± 1.08 (GB2), and 119.57 ± 1.13 (GB3) mg/kg (Figure 3a), respectively, which were all lower than the acceptable level of PCD's residential soil quality standard (400 mg/kg). This result is consistent with several previous findings (Phenrat et al., 2016; Poopa et al., 2015) reporting that uncontaminated soil samples in both the Upper and the Lower Klity areas are safe for human occupation. However, as shown in Figure 3b, the Pb concentration in the local Zn‐Pb deposit area was 111,759.09 ± 8,674.73 mg/kg, much higher than the Pb concentrations in uncontaminated soil, confirming that the local Zn‐Pb deposit is an elevated, natural source of Pb in the Lower Klity area.

#### 3.1.2 Pb Content in Samples From Tailing Storage Ponds

**Figure 4:**
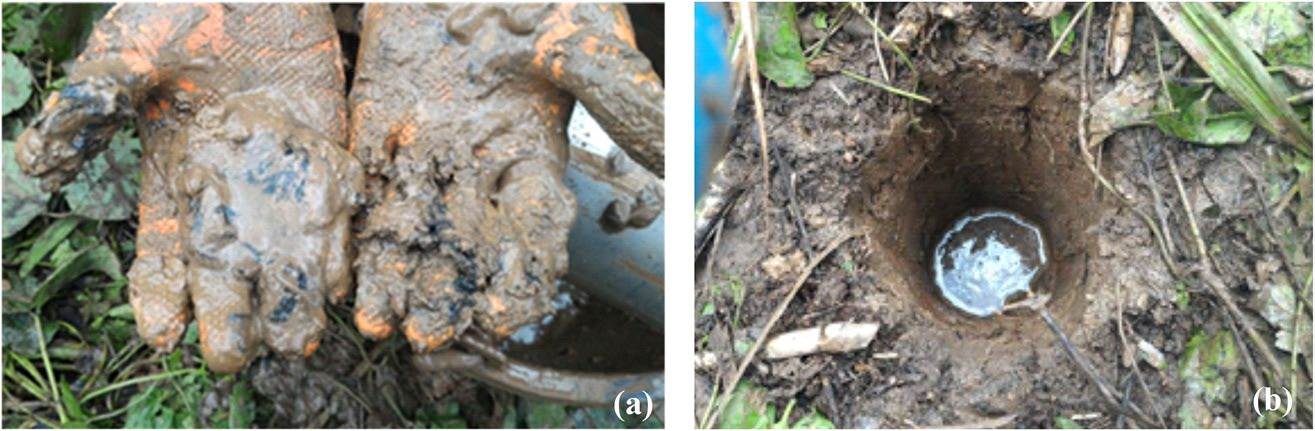
(a) The unnaturally very fine black particles in KT3P samples similar to the mine tailings from the ore flotation process, and (b) KT3P filled with water.

Figure 3b illustrates the Pb concentrations of samples inside KT2P and KT3P as a function of depth. As expected, the Pb concentrations in some samples were much higher than the acceptable level of PCD's residential soil quality standard (400 mg/kg). The highest Pb concentration in soil samples from KT2P was 24,544.07 ± 816.59 mg/kg (150–175 cm deep), while the highest Pb concentrations in soil samples from KT3P was 386,199.42 ± 9,349.61 mg/kg (175–200 cm deep). The Pb concentrations of shallow samples (<150 cm deep) in KT2P and KT3P ranged from 80.32 ± 10.19 to 449.21 ± 4.26 mg/kg. Notably, we found that the samples with high Pb concentrations (from 11,542.38 ± 279.19 to 386,119.4 2 ± 9,349.61 mg/kg) were the ones containing the unnaturally very fine black particles (Figure 4a) similar to the mine tailings from the ore flotation process. The high Pb concentrations were usually found at depths of 150–300 cm from the surface of the tailing ponds. This result is consistent with the PCD report, which declared that after the Klity ore dressing facility was shut down, the tailing storage ponds were covered by natural soil ∼150 cm thick (PCD, 2014). Also, the high Pb concentration in the mine tailing samples in this study is consistent with the high Pb concentration in the mine tailings sampled by PCD in 2010. Specifically, in 2010, the Pb concentration ranged from 29,041 to 161,500 mg/kg at a depth of 1.45–5.45 m below the surface, likely because the mine tailings are residual from dressing Pb crude ore consisting of galena (PbS), cerussite (PbCO_3_), and some anglesite (PbSO_4_) in limestone, clay, and quartz matrices (Phenrat et al., 2016). Thus, considering their high Pb concentration, depth, and physical appearance, mine tailing samples in this study can be positively identified.

Notably, during our field sampling, the KT3P and KT2P were filled with water (Figure 4b), presumably from the natural reservoir located at a higher elevation. Even with coefficients of soil permeability of <8.64 × 10^−5^ m/day (PCD, 2014), the water from the reservoir can easily migrate through the KT2P and KT3P. This is an important issue because natural water seeping from the natural reservoir to the KT2P and KT3P can promote continuous leaching of Pb from the mine tailings into Klity Creek. This hypothesis is evident by detecting seepage from the tailing ponds to the creek in 2018 to be discussed next.

#### 3.1.3 Pb Content in the Sediment of Klity Creek

Figure 3c illustrates the Pb concentration in the sediment samples collected from the different stations in Klity Creek (KC2, KC3, KC4, KC4/1, and KC5) downstream from the tailing ponds. The Pb concentrations in these samples were higher than in the upstream sediment (KC1 = 304.39 ± 11.69 mg/kg) by a factor of almost 1,500. The highest concentration was found at KC3 followed by KC4, KC5, KC2, and KC4/1 (459,056.41 ± 10,323.58 mg/kg, 352,162.24 ± 5,551.11 mg/kg, 163,273.29 ± 4,483.286 mg/kg, 140,210.30 ± 2,947.05 mg/kg, and 6,954.673 ± 401.55 mg/kg, respectively). The Pb concentrations in these contaminated sediment samples were higher than the acceptable sediment quality standard (130 mg/kg) and were much higher than the Pb concentrations in uncontaminated soil samples in the area (74.74 ± 1.08–294.40 ± 12.18 mg/kg). The elevated Pb concentrations in the Klity sediment samples are consistent with previous studies for which samples were collected in 2014 (KC2 = 51,008.82 ± 42,512.21 mg/kg, KC4 = 42,608.33 ± 17,619.69 mg/kg, KC4/1 = 34,145.00 ± 12,883.79 mg/kg, KC5 = 21,358.79 ± 18,926.72 mg/kg) (PCD, 2010; Phenrat et al., 2016). Furthermore, the Pb concentration increased at the same sampling stations (for example, KC2, KC4, and KC5) from 2014 to 2016. These results suggest that the natural attenuation of Klity Creek is ineffective in restoring the bottom sediment. The Pb increase resulted from the potential addition and migration of Pb from the mine tailing pond or the flood‐induced release of Pb‐contaminated sediment trapped by two rock‐check dams in the rainy season (Phenrat et al., 2016).

### 3.2 Pb Source Apportionment by Stable Pb Isotope Ratios

The stable Pb isotope ratios of all mine tailings, background soil, and sediment samples in this study are available in Table S4 in SI. Several findings for each group of samples are summarized here for source apportionment analyses.

#### 3.2.1 Isotope Fingerprinting of Mine Tailing and Geological Background

**Figure 5:**
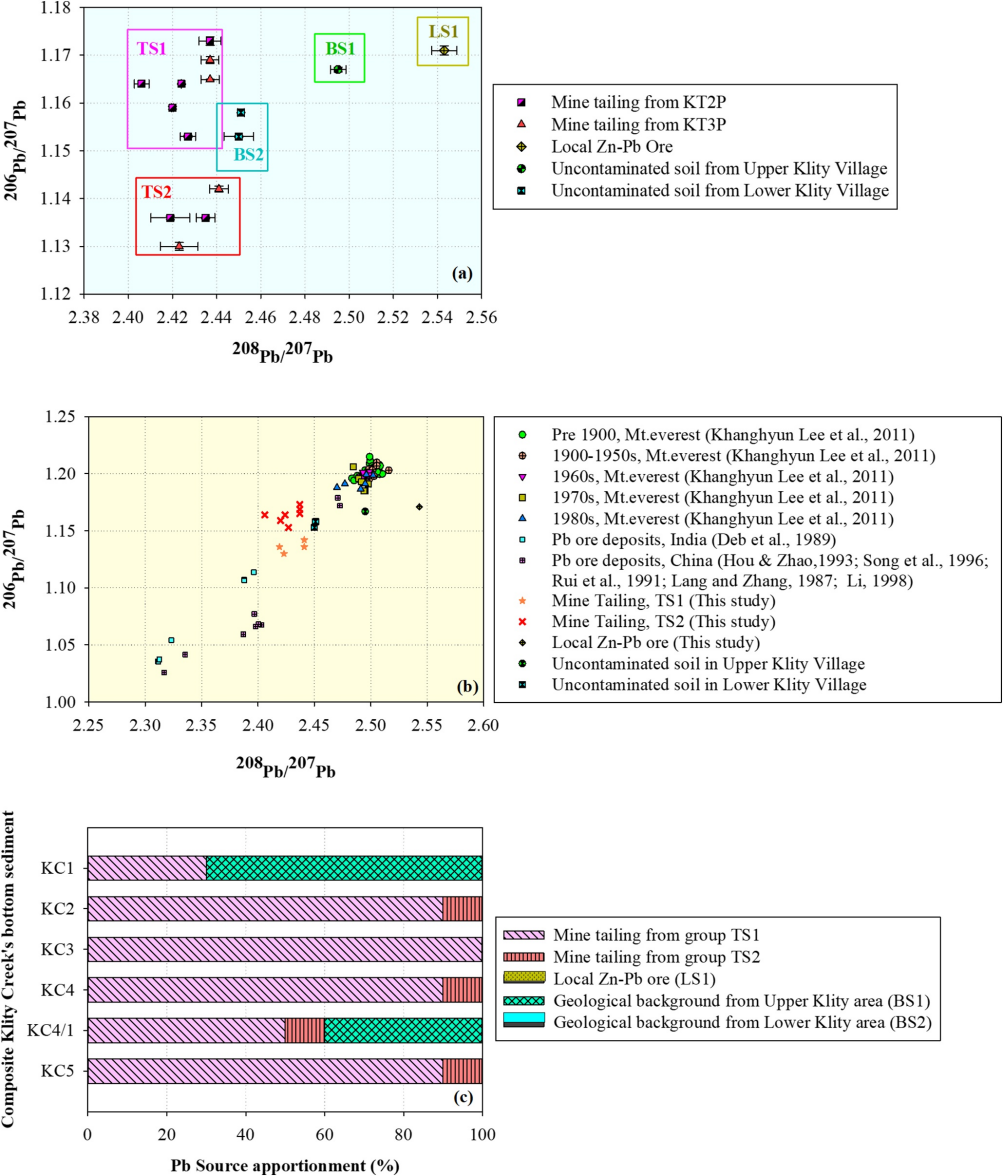
(a) Isotope fingerprinting of mine tailing and geological background, (b) the comparison of isotope Pb ratios (^206^Pb/^207^Pb and ^208^Pb/^207^Pb) of mine tailing and geological background in this study with other studies, and (c) results of Pb source apportionment in sediment samples from Klity Creek.

The objective of this section was to identify the two end‐members (i.e., the mine tailing and geological background) for further source apportionment. The mine tailing samples from KT2P at a depth of 25–50 cm and 150–175 cm as well as from KT3P at a depth of 150–175 cm, 175–200 cm, and 275–300 cm were selected to represent the mine tailings because their Pb concentration, location, and physical characteristics were similar to previously reported Pb‐ore tailings in the flotation process (PCD, 2010). As shown in Figure 5a, the stable Pb isotope ratios of these mine tailing samples show two distinct zones. The first zone, called TS1, was 1.130–1.142 for ^206^Pb/^207^Pb and 2.419–2.441 for ^208^Pb/^207^Pb. The second zone, called TS2, was 1.153–1.173 for ^206^Pb/^207^Pb and 2.406–2.437 for ^208^Pb/^207^Pb. When plotting the Pb stable isotope results of some other samples (with lower Pb concentrations) collected from the tailing ponds in the same graph, they fell into the two zones. Both zones consist of samples from KT2P and KT3P, suggesting that the mine tailing samples may be from two different geological sources and thus have two distinct isotope characteristics.

Since the tailings are residual from dressing Pb crude ore of the Bo Ngam mine, they should demonstrate the unique Pb stable isotope characteristics of the mine ore. The Bo Ngam ore is classified as carbonate‐hosted base metal sulfide deposits, including Mississippi Valley‐type (MVT) and sedimentary‐exhalative (SEDEX) deposits (PCD, 2014). The MVT deposits, named after three famous base metal districts in the United States (Sangster et al., 2000), occur worldwide and comprise sphalerite and galena in carbonate rock strata (Sangster et al., 2000). In contrast, SEDEX deposits are shale‐hosted Zn‐Pb‐Ag massive sulfides or clastic‐dominated Zn‐Pb deposits (Sangster et al., 2000). Previous X‐ray diffraction analysis confirmed the mine tailings consist of galena (PbS), cerussite (PbCO_3_), and some anglesite (PbSO_4_) in limestone, clay, and quartz matrices (Sangster et al., 2000). For this reason, we compared the Pb isotope ratios of the mine tailings in this study with those from the MVT and SEDEX in previous studies (Figure S1). We found that the Pb isotope ratios of TS1 were similar to MVT and SEDEX types of Argentina and Spain, which range from 1.146 to 1.154 for ^206^Pb/^207^Pb and 2.431 to 2.436 for ^208^Pb/^207^Pb (Gemmel et al., 1992; Macfarlane et al., 1990; Tornos & Arias, 1993). In contrast, the Pb isotope ratios of TS2 were similar to MVT and SEDEX of Argentina, Canada, Spain, and Norway, which range from 1.146 to 1.192 for ^206^Pb/^207^Pb and 2.404 to 2.437 for 208Pb/207Pb (Bjorlykke et al., 1993; Gemmel et al., 1992; Godwin et al., 1988; Macfarlane et al., 1990; Tornos & Arias, 1993).

Similarly, the stable Pb isotope ratios for the geological background samples fell into two distinct groups, BS1 for the Upper Klity village and BS2 for the Lower Klity village (Figure 5a). The ranges of the stable isotopes of BS1 were 1.158–1.176 for ^206^Pb/^207^Pb and 2.486–2.504 for ^208^Pb/^207^Pb while the ranges of stable isotopes of BS2 were 1.153–1.158 for ^206^Pb/^207^Pb and 2.450–2.451 for ^208^Pb/^207^Pb. The stable Pb isotope ratio for the local Zn‐Pb deposit (LS1) was unique, that is 1.165–1.177 for ^206^Pb/^207^Pb and 2.530–2.556 for ^208^Pb/^207^Pb. According to Figure S1, we found that the Pb isotope ratios of LS1 were similar to MVT and SEDEX types of Australia, Belgium, Canada, and Norway, which range from 1.160 to 1.179 for ^206^Pb/^207^Pb and 2.519 to 2.529 for ^208^Pb/^207^Pb (Bjorlykke et al., 1993; Coron, 1981; Cumming et al., 1990; Godwin et al., 1982, 1988; Godwin & Sinclair, 1982; Höy & Godwin, 1988; LeCouteur & Clifford, 1973; Léon, 1998; McCracken, 1997; Sangster & Vaillancourt, 1990; Shanks et al., 1987).

Thus, from these analyses, we obtained five distinct stable isotope ratios representing two mine tailing zones, two natural background zones, and a local source zone. The stable Pb isotope ratios of mine tailings and geological background in this study were consistent with previous studies, that is, the ranges of Pb isotope ratio values of the geological background were always higher than those of the mine tailings (Choi et al., 2013; Lee et al., 2011; Margui et al., 2006; Sangster et al., 2000; Stacey & Kramers, 1975; Wong et al., 2002; Zhu et al., 2001) (see also Figure 5b to compare the present study with previous studies). The stable Pb isotope ratios, as well as total Pb concentration of the samples assigned as mine tailing and geological background soil, are summarized in Table 2.

**Table 2:** The Stable Pb Isotope Ratios and Pb Concentration of Mine Tailing and Geological Background

Source of Pb	Stable Pb isotope ratios		Pb concentration (mg/kg)
	^206^Pb/^207^Pb	^208^Pb/^207^Pb	
**TS1**	1.130–1.142	2.419–2.441	279.331 ± 5.54–386,199.417 ± 9,349.61
**TS2**	1.153–1.173	2.406–2.437	259.024 ± 6.28–112,626.882 ± 1,179.15
**LS1**	1.165–1.177	2.530–2.556	111,759.09 ± 8,674.73
**BS1**	1.158–1.176	2.486–2.504	294.404 ± 12.18
**BS2**	1.153–1.158	2.450–2.451	74.743 ± 1.08–119.572 ± 1.13

#### 3.2.2 Source Apportionment Based on Pb Isotope Ratios and Pb Concentration

Now that we have the stable Pb isotope ratios and Pb concentration characteristics of the two end members (but consisting of five distinct members), we can apply the source apportionment analysis, based on Equations (1), (2), (3), (4), (5) , for Klity Creek's sediment samples. The Pb stable isotope ratios of KC1 to KC5 are illustrated in Table S4. The optimized results for each sediment sample are summarized in Table S5, together with the statistics of the fitting. Figure 5c illustrates the source apportionment of Pb in sediment from the Klity Creek from upstream to downstream. As shown in Figure 5c, the mine tailings from TS1, accounting for 30%–100% of the total Pb contamination in the sediment samples, was the primary source of Pb contamination in Klity Creek. The mine tailings from TS2 accounted for 0%–10% of the total Pb contamination in the sediment samples, making it the secondary source of Pb contamination in Klity Creek. Only 40%–70% was from the geological background, BS1, while no substantial contribution was from BS2. The BS1 from the Upper Klity area can affect KC1 to KC4, while BS2 in the Lower Klity area can potentially contribute to only the sediment samples at KC5. Nevertheless, from the source apportionment analyses, BS2 does not substantially contribute to the Pb in Klity Creek's sediment at KC5.

There were several notable trends from the source apportionment analyses. First, even at KC1, upstream of the spill point, 30% of Pb was attributed to mine tailing. This unexpected finding can be explained by the fact the mine illegally dumped mine tailings in several locations along the flood plains, agricultural areas, and in residential areas. More than 30 locations of illegal dumping of mine tailings, some of which were upstream of the spill point, were discovered by PCD and villagers (PCD, 2010, 2014). This substantial contribution from mine tailings at KC1 may be a result of some of these illegal dumping sites. As expected, the contribution from mine tailings is very high at KC2, KC3, and KC4. Notably, KC4/1 had a much higher contribution from the natural background than KC4. Presumably, since KC4/1 is the second rock‐check dam in series, large mine tailings that flowed or rolled through the creek may have been trapped at KC4, and a smaller portion of the tailings could have been trapped at KC4/1. This makes the mine tailing contribution smaller relative to the natural background at KC4/1. This hypothesis is also supported by the lowest Pb concentration at KC4/1 (6,954.673 ± 401.55 mg/kg) in comparison to Pb concentrations in the sediment at the other monitoring stations (140,210.30 ± 2,947.05 mg/kg at KC2 and 459,056.41 ± 10,323.58 mg/kg at KC3). These results suggest that sediment samples at KC4/1 were greatly diluted with natural background sediment that had a low Pb concentration.

The finding on the major contamination contribution from tailing ponds to Klity Creek is consistent with not only historical Pb‐mine‐tailing spills but also the assumption of continuous leakage of Pb from tailing ponds. Nevertheless, using stable Pb isotope in this section cannot distinguish if the Pb contamination from the tailing ponds is from the past spills or the current leakage from the abandoned tailing ponds. This issue will be addressed in the next section.

### 3.3 Age of Sediment Samples by Radioactive Isotope ^210^Pb

**Figure 6:**
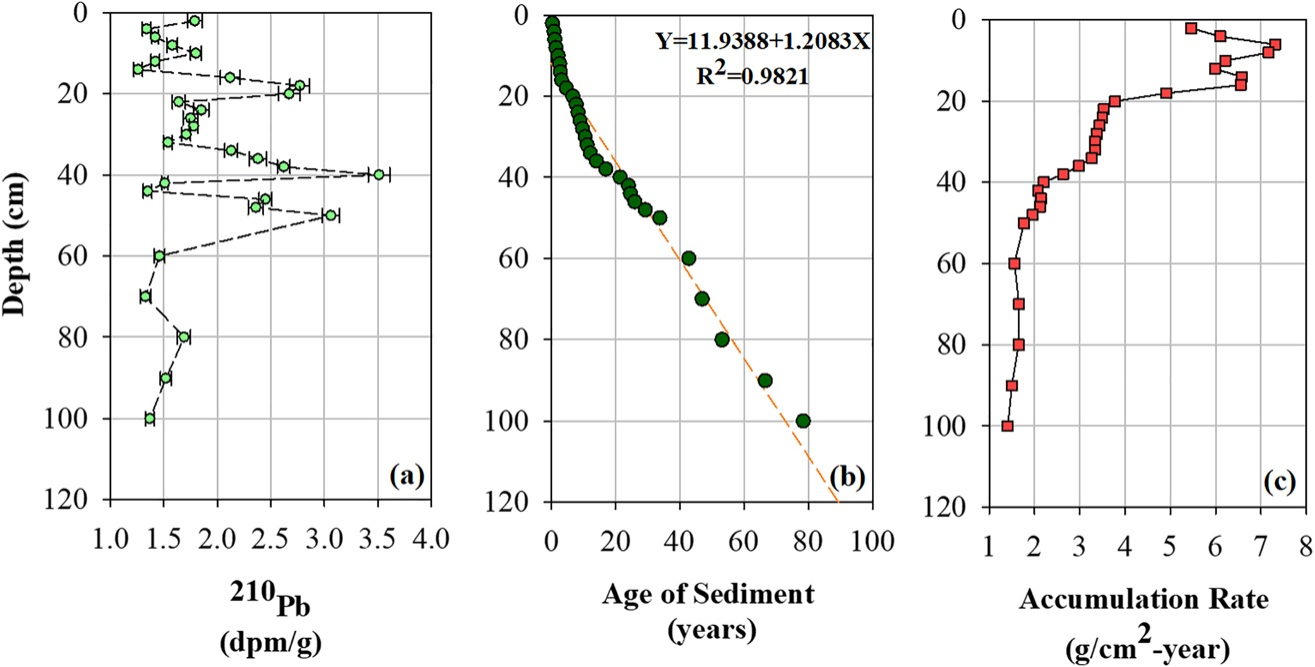
(a) Depth profile of excess ^210^Pb, (b) age of sediment at different depths using the constant rate of supply (CRS) model, and (c) sediment accumulation rates versus sediment depth.

Figure 6a shows the excess ^210^Pb profile as a function of depth. Notably, it did not decrease exponentially with depth, indicating that the sedimentation rates were not constant over time (Lubis, 2006). The excess ^210^Pb fluctuated from 0 to 60 cm deep then reached lower values at 100 cm deep. Using the CSR model, the bottom sediment samples in this study were dated between 0 and 80 years old (Figure 6b). Bottom sediments at 0–20 cm deep were dated 0–6.7 years, whereas sediments 0–40 cm, 40–60 cm, and 60–100 cm deep were dated 7.75–21.4 years, 24.06–42.83 years, and 42.83–78.45 years, respectively. The age of Pb‐contaminated sediments at shallow levels (0–20 cm) was relatively young, that is 0–6.7 years. However, they contained high Pb concentrations coupled with stable Pb isotope ratios similar to those of the mine tailings. The sediment accumulation rates at depth for KC5 support these results. Noticeably, the profile is shifted at depths of 0–18 cm, 20–40 cm, and 42–100 cm for the corresponding accumulation rates of 4.91–7.31 g/cm^2^‐year, 2.21–3.78 g/cm^2^‐year, and 1.41–2.21 g/cm^2^‐year, respectively (Figure 6c). The accumulation rates increased over time, from minimum values 100 years ago (see the match of sediment age and depth in Figure 6b) to the maximum values at present. Consequently, these multiple lines of evidence, including total Pb concentration, stable Pb isotope ratios, and sediment dating, suggest that the source of Pb contamination in Klity Creek (using KC5 as an example) was a recent Pb leakage from the tailing ponds rather than the remains of the mine tailing spill into the creek 15 years ago.

### 3.4 Pb Concentration in Seepage and Groundwater Samples

Pb concentrations in seepage samples along the edge of KT2P and KT3P were 0.1–0.53 mg/L (with the average of 0.30 ± 0.22 mg/L), which is 23 times higher than the Pb concentration at Dee Ka Creek (0.013 ± 0.017 mg/L), upstream of the tailing ponds. This result suggests that water flowing from the natural reservoir through the tailing ponds is leaching Pb out of mine tailing. This Pb‐contaminated leachate becomes seepage, which migrates Pb to the Klity Creek.

Furthermore, as shown in Table 3, Pb concentration in groundwater samples from the monitoring well for KT1P, an empty tailing pond, was substantially lower than that of the monitoring wells for KT2P and KT3P. For example, the total and dissolved Pb concentrations in groundwater samples from GW3 installed to monitor KT1P, were the lowest, 0.029 and 0.018 mg/L, respectively, while the total and dissolved Pb concentrations in groundwater samples from GW4 to monitor upgradient of KT3P were 0.141 and 0.077 mg/L, respectively. Furthermore, the total and dissolved Pb concentrations in groundwater samples from GW2 to monitor downgradient of KT3P were the highest, 0.225 and 0.079 mg/L, respectively. As noted above, the shallow groundwater samples from these monitoring wells were ∼6–17 times greater than the Pb concentration at Dee Ka Creek (0.013 ± 0.017 mg/L), upstream of the tailing ponds. Moreover, the Pb concentrations in the groundwater sample from GW2 (collected in 2020) close to the seepage location were similar to the Pb concentrations in seepage in 2018 (i.e., 0.225 mg/L for GW2 and 0.30 ± 0.22 mg/L for seepage). This result confirms the findings from the stable isotope results that KT2P and KT3P are leaking Pb to Klity Creek. Although some portion of Pb is released from the tailing ponds as dissolved species, the dissolved Pb can undergo surface complexation and precipitation with carbonate species in the creek to form Pb‐contaminated precipitate or sediment as observed in the field.

**Table 3:** Total and Dissolved Pb Concentrations in Groundwater Monitoring Wells Surrounding Tailing Ponds

Location	Pb (mg/L)	
	Total	Dissolved
GW1	0.034	0.017
GW2	0.225	0.079
GW3	0.029	0.018
GW4	0.141	0.077

## 4 Conclusions and Implementation

Four lines of evidence were used to systematically assess if the leakage from tailing ponds is primarily responsible for the long‐term Pb contamination in Klity Creek. First, stable Pb isotope ratios (^206^Pb/^207^Pb and ^208^Pb/^207^Pb) were used to apportion sources between the tailing and geological background. With this first approach, we found that the mine tailing contributed from 30% to 100% of the total Pb contamination in the sediment samples in Klity Creek. On the other hand, only 40%–70% was from the geological background. Second, the analysis of ^210^Pb was used to investigate whether the Pb in the contaminated sediment was relatively new, as if from recent leakage from the tailing storage ponds, or is relatively old as if from the 15‐year‐old tailing spill. With this approach, we found that the age of Pb‐contaminated sediment at KC5 at the depth of our investigation (0–20 cm) was relatively young, that is 0–6.7 years, but they had high Pb concentration coupled with stable Pb isotope ratios in the range of the mine tailings. Thus, the source of Pb contamination in the Klity Creek (using KC5 as an example) was a recent Pb leakage from the tailing ponds rather than the remaining of the mine tailing spill into the creek 15 years ago.

Two last two lines of evidence were the Pb concentrations in observed seepage samples and groundwater samples from the groundwater monitoring wells surrounding the tailing ponds. We found that the Pb concentrations in the seepage from the tailing ponds and groundwater samples from monitoring wells surrounding the tailing ponds were 0.30 ± 0.22 mg/L and 0.029–0.225 mg/L, respectively. These contaminations were 6–23 times higher than the Pb concentration at Dee Ka Creek (0.013 ± 0.017 mg/L), upstream of the tailing ponds. This result suggests that water flowing from the natural reservoir through the tailing ponds is leaching Pb out of the mine tailings. This Pb‐contaminated leachate becomes seepage, which migrates Pb to the Klity Creek.

This study ends the 20‐year debate on the source of Pb contamination in the Klity Creek. It disproves the hypothesis that restoration of Klity Creek is impossible because of a too‐high natural Pb background in that area that continuously adds Pb to the creek by soil erosion. The findings in this study also provide a solid explanation for the poor natural recovery of Pb contamination in the Klity Creek (Phenrat et al., 2016). It shows clearly that the reservoir at the higher elevation of tailing ponds promotes the migration of Pb out of the tailing ponds. Thus, to achieve the remedial action goal ordered by the court, proper tailing ponds management is imperative. We recommend the PCD, responsible for Klity Creek restoration, spend a substantial portion of its restoration budget to investigate and cease the Pb leakage from the tailing ponds instead of spending all the budget to dredge the Pb‐contaminated sediment out of the Klity Creek because, after the expensive dredging operation, the tailing ponds may leak Pb back to the creek, delaying the site closure.

## Conflict of Interest

The authors declare no conflicts of interest relevant to this study.

## Supporting information

Supporting Information S1

## Data Availability

Datasets for this research are available in these in‐text data citation references: Phenrat, Tanapon (2020), “Lead (Pb) Isotope of Abandoned Mine Tailing Storage Ponds in Kanchanaburi Province, Thailand,” Mendeley Data, V2, http://doi.org/10.17632/hb2y9fppwj.2.
