# Supplementary material for: Assessment of Lead (Pb) Leakage From Abandoned Mine Tailing Ponds to Klity Creek, Kanchanaburi Province, Thailand
Source: Geohealth. 2021 May 1;5(5):e2020GH000252. doi: 10.1029/2020GH000252 (PMC8101536; doi:10.1029/2020GH000252)
Supplement: Supplementary file 1 — Supporting Information S1 [file GH2-5-e2020GH000252-s001.docx]

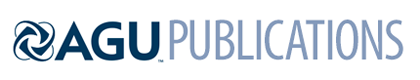


*GeoHealth*

Supporting Information for

**Assessment of Lead (Pb) Leakage from Abandoned Mine Tailing Ponds to Klity Creek, Kanchanaburi Province, Thailand**

Supawan Srirattana^1,2,3^, Kitsanateen Piaowan^2,3^, Thanyathit Imthieang^3^,
Jiraporn Suk-in^2,3^, and Tanapon Phenrat^1,2,3*^

^1^Department of Civil Engineering, Faculty of Engineering, Naresuan University, Phitsanulok, Thailand, 65000.

^2^Research Unit for Integrated Natural Resources Remediation and Reclamation (IN3R) Department of Civil Engineering, Faculty of Engineering, Naresuan University, Phitsanulok 65000, Thailand.

^3^Center of Excellence for Sustainability of Health, Environment and Industry (SHEI), Faculty of Engineering, Naresuan University, Phitsanulok 65000, Thailand.

**Contents of this file**

Figures S1. The comparison of Pb isotope ratios (^206^Pb/^207^Pb and ^208^Pb/^207^Pb) of mine tailing and Local Zn-Pb ore in this study with world MVT and SEDEX Pb ore deposits

Figures S2. NIST SRM 981 Pb standard working data (^204^Pb ^206^Pb ^207^Pb and ^208^Pb) from Agilent ICP-MS MassHunter Software.

Figures S3. NIST SRM 981 Pb standard working curve (^204^Pb ^206^Pb ^207^Pb and ^208^Pb)

Figures S4. Internal standard recovery of ^209^Bi at 1 µg/L

Table S1. Mean (+S.D.) Pb isotope ratios of world MVT and SEDEX Pb ore deposits

Table S2. ICP-MS instrument operating conditions (Agilent 7900, Agilent, Santa Clara, US)

Table S3. NIST SRM 981 Pb standard working data (^204^Pb ^206^Pb ^207^Pb and ^208^Pb)

Table S4. Pb Content and Stable Pb isotope ratios in Mine Tailing, Local Zn-Pb Deposit, Uncontaminated Geological Background Soil, and Sediment Sampling in 2016

Table S5. Source apportionment based on Pb isotope ratios and Pb concentration

Isotopic composition of Pb ore deposits Stable Pb isotope data were compiled from the geological literature for 101 ore deposits (862 ore samples) (Table S1).

**Table S1. Mean (+S.D.) Pb isotope ratios of world MVT and SEDEX Pb ore deposits**

| **Country** | **Deposit/District** | **N** | **^206^Pb/^207^Pb** | **^208^Pb/^207^Pb** | **Reference** |
| --- | --- | --- | --- | --- | --- |
| Alaska | Red Dog | 1 | 1.1805±0.0004 | 2.4512±0.0029 | (Gulson, 1986; Lange et al., 1985) |
| Argentina | Aguilar | 21 | 1.1535±0.0006 | 2.4313±0.0011 | (Gemmel et al., 1992; Macfarlane et al., 1990; Puig, 1990) |
|  | Esperanza | 5 | 1.1530±0.0005 | 2.4316±0.0013 | (Gemmel et al., 1992) |
| Australia | Abra | 1 | 1.0313 | 2.3086 | (Cumming & Richards, 1975) |
|  | Admiral Bay | 12 | 1.1653±0.0029 | 2.4549±0.0033 | (McCracken et al., 1997) |
|  | Blendvale | 7 | 1.2257±0.0043 | 2.5187±0.0051 | (Vearncombe et al., 1995) |
|  | Broken Hill | 30 | 1.0407±0.0006 | 2.3153±0.0013 | (Gulson, 1984) |
|  | Cadjebut | 17 | 1.2193±0.0009 | 2.5007±0.0015 | (Tompkins et al., 1994) |
|  | Coxco | 31 | 1.0482±0.0045 | 2.3209±0.0037 | (Vaasjoki & Gulson, 1986; Walker et al., 1983) |
|  | Dugald River | 8 | 1.0434±0.0036 | 2.3177±0.0008 | (Gulson, 1985) |
|  | HYC | 12 | 1.0449±0.0005 | 2.3179±0.0008 | (Gulson, 1985) |
|  | Hellyer | 13 | 1.1757±0.0007 | 2.4456±0.0012 | (Gulson, 1986) |
|  | Hilton | 6 | 1.0431±0.0005 | 2.3177±0.0004 | (Gulson, 1985) |
|  | Lady Loretta | 28 | 1.0493±0.0024 | 2.3217±0.0018 | (Gulson, 1985) |
|  | Mount Isa | 14 | 1.0431±0.0005 | 2.3181±0.0008 | (Gulson, 1985) |
|  | Que River | 15 | 1.1736±0.0008 | 2.4440±0.0011 | (Gulson, 1986) |
|  | Rosebery | 11 | 1.1714±0.0006 | 2.4404±0.0012 | (Gulson, 1986) |
|  | Sorby Hills | 15 | 1.2043±0.0014 | 2.4675±0.0012 | (Vaasjoki & Gulson, 1986) |
| Austria | Alpine | 6 | 1.1721±0.0011 | 2.4605±0.0012 | (Brigo et al., 1977; Zartman et al., 1979) |
|  | Gratzer Paleozoicum | 8 | 1.1514±0.0004 | 2.4400±0.0011 | (Koppel & Schroll, 1983) |
| Belgium | Dinant district | 10 | 2.4573±0.0084 | 2.0906±0.0059 | (Léon, 1998) |
|  | Veriers district | 16 | 2.4558±0.0029 | 2.0877±0.0026 | (Léon, 1998) |
| Canada | Big Ledge | 8 | 2.5293±0.0024 | 1.2306±0.0022 | (Godwin et al., 1988) |
|  | Cottonbelt | 13 | 2.4421±0.0018 | 1.177±0.0014 | (Godwin et al., 1988; Höy & Godwin, 1988) |
|  | DY | 43 | 2.4513 ±0.0053 | 1.1765 ±0.0020 | (Godwin & Sinclair, 1982; Shanks et al., 1987) |
|  | Driftpile | 3 | 2.4962 ±0.0027 | 1.2036 ±0.0011 | (Godwin & Sinclair, 1982; Godwin et al., 1982) |
|  | Duncan | 2 | 2.5247 | 1.2300 | (Godwin et al., 1988) |
|  | Faro | 12 | 2.4479 ±0.0025 | 1.1727 ±0.0013 | (Godwin & Sinclair, 1982; LeCouteur & Clifford, 1973) |
|  | Gayna River | 15 | 2.441 ±0.0197 | 1.188 ±0.0216 | (Godwin et al., 1988) |
|  | Gays River | 2 | 2.4452 | 1.1564 | (Akande & Zentilli, 1983; Sangster et al., 1998) |
|  | Grum | 7 | 2.4476 ±0.007 | 1.1751 ±0.0016 | (Godwin & Sinclair, 1982) |
|  | Howards Pass | 11 | 2.4676 ±0.0054 | 1.1877 ±0.0052 | (Godwin & Sinclair, 1982; Morganti, 1979) |
|  | HB | 4 | 2.5049 ±0.0086 | 1.2123 ±0.0019 | (Godwin et al., 1988) |
|  | Jackpot | 3 | 2.4955 ±0.0051 | 1.2062 ±0.0017 | (Godwin et al., 1988) |
|  | Jason | 5 | 2.4807 ±0.0304 | 1.1932 ±0.0025 | (Godwin & Sinclair, 1982) |
|  | Jersey | 6 | 2.5083 ±0.0075 | 1.2113 ±0.0018 | (Godwin et al., 1988) |
|  | Jordan River | 3 | 2.4327±0.0047 | 1.155±0.0110 | (Godwin et al., 1988) |
|  | Monarch /  Kicking Horse | 2 | 2.4776 | 1.1848 | (Godwin & Sinclair, 1982; Godwin et al., 1982) |
|  | Newfoundland  Zinc Mine | 9 | 2.493±0.0084 | 1.1603±0.0089 | (Coron, 1981) |
|  | Pine Point | 31 | 2.4528±0.0115 | 1.1671±0.0003 | (Cumming et al., 1990) |
|  | Polaris | 3 | 2.4505 ±0.0017 | 1.1482±0.0010 | (Heal, 1976) |
|  | Remac |  | 2.5029 ±0.0072 | 1.2096 ±0.0023 | (Godwin et al., 1988) |
|  | Robb Lake | 12 | 2.5867 ±0.0052 | 1.2736 ±0.0052 | (Godwin & Sinclair, 1982; Godwin et al., 1982; Morrow & Cumming, 1982) |
|  | Ruddock Creek | 3 | 2.4335 ±0.0028 | 1.1789 ±0.0009 | (Godwin et al., 1988) |
|  | Sullivan | 8 | 2.3376 ±0.0015 | 1.0679 ±0.0029 | (Godwin & Sinclair, 1982; LeCouteur & Clifford, 1973) |
|  | Swim | 6 | 2.4444 ±0.0021 | 1.1715 ±0.0008 | (LeCouteur & Clifford, 1973; Shanks et al., 1987) |
|  | Tom | 6 | 2.4692 ±0.0057 | 1.1909 ±0.0014 | (Godwin & Sinclair, 1982; Godwin et al., 1982) |
|  | Truro | 4 | 2.4500 ±0.0011 | 1.1513 ±0.0085 | (Heal, 1976) |
|  | Vangorda | 5 | 2.4447 ±0.0014 | 1.1718 ±0.0004 | (Godwin & Sinclair, 1982; LeCouteur & Clifford, 1973) |
|  | Vulcan | 11 | 2.4952 ±0.0220 | 1.1999 ±0.0033 | (Godwin & Sinclair, 1982) |
|  | Wigwam | 3 | 2.4466 ±0.0019 | 1.1656 ±0.0024 | (Godwin et al., 1988) |
|  | Wrigley | 16 | 2.5059 ±0.0110 | 1.2438 ±0.0121 | (Heal, 1976) |
|  | Yava | 11 | 2.4487 ±0.0029 | 1.1764 ±0.0026 | (Sangster & Vaillancourt, 1990) |
| India | Ambaji | 5 | 1.1072±0.0012 | 2.3876±0.0005 | (Deb et al., 1989) |
|  | Ambaji-Sendra belt | 12 | 1.1138±0.0215 | 2.3961±0.0255 | (Deb et al., 1989) |
|  | Deri | 4 | 1.1066±0.0002 | 2.3877±0.0009 | (Deb et al., 1989) |
|  | Rajpura | 4 | 1.0356±0.0002 | 2.3110±0.0009 | (Deb et al., 1989) |
|  | Rampura | 8 | 1.0354±0.0001 | 2.3114±0.0004 | (Deb et al., 1989) |
|  | Saladipura | 1 | 1.0372 | 2.3125 | (Deb et al., 1989) |
|  | Zawar | 15 | 1.0542±0.001 | 2.3231±0.001 | (Deb et al., 1989) |
| Japan | Kuroko district | 9 | 1.1822±0.0069 | 2.4784±0.0079 | (Sato & Sasaki, 1973) |
| China | Beishan | 1 | 1.0593 | 2.3869 | (Hou & Zhao, 1993) |
|  | Binggou | 4 | 1.0660±0.0039 | 2.3978± 0.0106 | (Hou & Zhao, 1993) |
|  | Dongshan | 5 | 1.0676 ±0.0072 | 2.4029 ± 0.0042 | (Hou & Zhao, 1993) |
|  | Fankou | 15 | 1.1720 ± 0.0016 | 2.4726 ± 0.0031 | (Song et al., 1996) |
|  | Guanmenshan | 47 | 1.0259 ± 0.0469 | 2.3168 ± 0.0404 | (Rui et al., 1991) |
|  | Gudonggou | 6 | 1.0681±0.0055 | 2.4003±0.0061 | (Cheng & Hu, 2010) |
|  | Jiashenpan | 15 | 1.0415±0.0063 | 2.3354±0.0116 | (Lang & Zhang, 1987) |
|  | Jinding | 27 | 1.1789±0.0004 | 2.4707±0.0005 | (Li, 1998) |
|  | Lugou | 6 | 1.0661±0.0024 | 2.3978±0.0037 | (Hou & Zhao, 1993) |
|  | Wafanggou | 1 | 1.0771 | 2.3967 | (Hou & Zhao, 1993) |
| South Africa | Big Syn | 3 | 1.0745±0.0008 | 2.3547±0.0017 | (Koeppel, 1978) |
|  | Black Mountain | 5 | 1.0762±0.0014 | 2.3543±0.0026 | (Koeppel, 1978) |
|  | Broken Hill | 7 | 1.0745±0.0016 | 2.3539±0.0021 | (Koeppel, 1978) |
|  | Gamsberg | 5 | 1.0775±0.0009 | 2.3536±0.0018 | (Koeppel, 1978) |
|  | Rozynen | 3 | 1.0905±0.0004 | 2.3556±0.0026 | (Koeppel, 1978) |
| Mexico | Encantada | 1 | 1.2012 | 2.4787 | (James & Henry, 1993) |
|  | Fresnillo | 9 | 1.2040±0.0010 | 2.4778±0.0017 | (Cumming et al., 1979) |
|  | Los Lamentos | 1 | 1.1978 | 2.4728 | (James & Henry, 1993) |
|  | Naica | 1 | 1.196 | 2.4654 | (James & Henry, 1993) |
|  | Ojuela | 1 | 1.1961 | 2.4702 | (Cumming et al., 1979) |
|  | Parral | 3 | 1.2031±0.0004 | 2.4742±0.0005 | (Cumming et al., 1979) |
|  | San Carlos | 3 | 1.1800±0.0098 | 2.4518±0.0103 | (James & Henry, 1993) |
|  | Santa Eulalia | 8 | 1.1867±0.0008 | 2.4606±0.0012 | (Cumming et al., 1979; James & Henry, 1993) |
|  | Taxco | 5 | 1.1980±0.0006 | 2.4736±0.0009 | (Cumming et al., 1979) |
|  | Velardena | 2 | 1.2051 | 2.4745 | (Cumming et al., 1979) |
|  | Zacatecas | 1 | 1.2044 | 2.4766 | (Cumming et al., 1979) |
| Spain | Betic Cordillera  district | 17 | 1.1878±0.0118 | 2.4795±0.0138 | (Arribas & Tosdal, 1994) |
|  | Reocin | 3 | 1.1951±0.0002 | 2.4711±0.0006 | (Velasco et al., 1996) |
|  | Rubiales | 7 | 1.1463±0.0010 | 2.4360±0.0011 | (Tornos & Arias, 1993) |
|  | Troya | 6 | 1.1875±0.0009 | 2.4722±0.0031 | (Velasco et al., 1996) |
| Norway | Bleikvassli | 5 | 1.1833±0.0004 | 2.4430±0.0003 | (Bjorlykke et al., 1993) |
|  | Gamvik | 2 | 1.1344 | 2.3932 | (Bjorlykke et al., 1993) |
|  | Malmhaugen | 3 | 1.1915±0.0014 | 2.4374±0.0034 | (Bjorlykke et al., 1993) |
|  | Mofjellet | 3 | 1.1663±0.0000 | 2.4350±0.0002 | (Bjorlykke et al., 1993) |
|  | Mosbergvik | 4 | 1.1590±0.0005 | 2.4243±0.0016 | (Bjorlykke et al., 1993) |
|  | Nonfjellet | 4 | 1.1512±0.0011 | 2.4037±0.0007 | (Bjorlykke et al., 1993) |
|  | Osen | 6 | 1.2785±0.0077 | 2.4846±0.0017 | (Köppel & Schroll, 1988) |
|  | Skarnesdal | 5 | 1.1333±0.0030 | 2.3935±0.0025 | (Bjorlykke et al., 1993) |

**Figures S1. The comparison of Pb isotope ratios (^206^Pb/^207^Pb and ^208^Pb/^207^Pb) of mine tailing and Local Zn-Pb ore in this study with world MVT and SEDEX Pb ore deposits.**

Table S2. ICP-MS instrument operating conditions (Agilent 7900, Agilent, Santa Clara, US)

| **Instrument Parameter** | **Value** |
| --- | --- |
| RF power | 1550 W |
| RF Matching | 1.70 V |
| Smpl Depth | 10.0 mm |
| Carrier Gas | 0.75 L/min |
| Nebulizer Pump | 0.10 rps |
| S/C Temp | 2 ^o^C |
| Gas Switch | Makeup Gas |
| Makeup/Dilution Gas | 0.25 L/min |
| Integration Time | 0.1 sec |
| Sampling Period | 0.311 sec |

Table S3. NIST SRM 981 Pb standard working data (^204^Pb ^206^Pb ^207^Pb and ^208^Pb)

| ID | ^204^Pb | | | ^206^Pb | | |
| --- | --- | --- | --- | --- | --- | --- |
|  | Conc. [ppb] | Conc. RSD | CPS | Conc. [ppb] | Conc. RSD | CPS |
| STD 0 ppb | <0.000 | N/A | 577.8633333 | <0.000 | N/A | 9348.97 |
| STD 5 ppb | 0.132322784 | 0.934552893 | 32298.82333 | 2.28117838 | 0.762464208 | 558010.5433 |
| STD 10 ppb | 0.273332056 | 2.800251385 | 63360.06667 | 4.697374282 | 0.798703355 | 1088566.783 |
| STD 25 ppb | 0.670724177 | 0.625164541 | 149949.53 | 11.59361344 | 0.423546724 | 2587227.803 |
| STD 50 ppb | 1.338741182 | 1.274468433 | 303167.37 | 22.96061587 | 0.840017717 | 5185984.893 |
| ID | ^207^Pb | | | ^208^Pb | | |
|  | Conc. [ppb] | Conc. RSD | CPS | Conc. [ppb] | Conc. RSD | CPS |
| STD 0 ppb | <0.000 | N/A | 8014.566667 | <0.000 | N/A | 20158.77 |
| STD 5 ppb | 2.090054058 | 1.655403517 | 514296.8767 | 4.927758625 | 0.742353862 | 1216104.323 |
| STD 10 ppb | 4.310931248 | 0.164570053 | 1002728.373 | 10.2423493 | 0.716467838 | 2393863.16 |
| STD 25 ppb | 10.66290017 | 0.268689868 | 2385291.577 | 25.1897646 | 0.374508776 | 5670005.28 |
| STD 50 ppb | 21.04234156 | 0.42165118 | 4762723.833 | 49.86387198 | 0.622305429 | 11361958.82 |

**Table S4. Pb Content and Stable Pb isotope ratios in Mine Tailing, Local Zn-Pb Deposit, Uncontaminated Geological Background Soil, and Sediment Sampling in 2016**

| No. | ID | Pb  (mg/kg) | Stable Pb isotope ratios | |
| --- | --- | --- | --- | --- |
|  |  |  | ^206^Pb/^207^Pb | ^208^Pb/^207^Pb |
| Composite Klity Creek’s bottom sediment | | | | |
| 1 | KC1 | 304.389 ± 11.69 | 1.148 ± 9.179E-04 | 2.486 ± 9.446E-03 |
| 2 | KC2 | 140,210.302 ± 2,947.05 | 1.135 ± 4.947E-04 | 2.425 ± 4.488E-04 |
| 3 | KC3 | 459,056.411 ± 10,323.58 | 1.144 ± 7.702E-04 | 2.482 ± 5.458E-03 |
| 4 | KC4 | 352,162.242 ± 5,551.11 | 1.132 ± 4.402E-06 | 2.416 ± 1.993E-03 |
| 5 | KC4/1 | 6,954.673 ± 401.55 | 1.139 ± 2.597E-03 | 2.476 ± 1.635E-02 |
| 6 | KC5 | 163,273.286 ± 4,483.286 | 1.130 ± 6.942E-05 | 2.417 ± 2.832E-03 |
| Mine tailing samples | | | | |
| 7 | KT2P:0-5 | 288.561 ± 5.78 | 1.173 ± 8.790E-04 | 2.437 ± 4.973E-03 |
| 8 | KT2P:5-25 | 449.206 ± 4.26 | 1.159 ± 2.937E-04 | 2.420 ± 4.550E-04 |
| 9 | KT2P:25-50 | 11,542.382 ± 279.19 | 1.164 ± 5.772E-08 | 2.406 ± 3.357E-03 |
| 10 | KT2P:75-100 | 370.55 ± 23.79 | 1.136 ± 3.726E-04 | 2.419 ± 8.843E-03 |
| 11 | KT2P:100-125 | 279.331 ± 5.54 | 1.136 ± 1.724E-04 | 2.435 ± 4.188E-03 |
| 12 | KT2P:125-150 | 426.283 ± 3.31 | 1.164 ± 8.523E-05 | 2.424 ± 6.852E-05 |
| 13 | KT2P:150-175 | 24,544.067 ± 816.59 | 1.153 ± 2.416E-04 | 2.427 ± 3.433E-03 |
| 14 | KT3P:0-5 | 259.024 ± 6.28 | 1.165 ± 1.215E-04 | 2.437 ± 4.114E-03 |
| 15 | KT3P:5-25 | 80.321 ± 10.19 | 1.189 ± 4.390E-04 | 2.640 ± 2.820E-02 |
| 16 | KT3P:25-50 | 139.144 ± 9.56 | 1.222 ± 4.764E-03 | 2.627 ± 5.118E-03 |
| 17 | KT3P:50-75 | 99.799 ± 5.93 | 1.181 ± 1.297E-03 | 2.619 ± 1.133E-02 |
| 18 | KT3P:75-100 | 491.344 ± 17.62 | 1.152 ± 4.627E-04 | 2.544 ± 3.306E-03 |
| 19 | KT3P:100-125 | 146.943 ± 21.16 | 1.150 ± 9.272E-04 | 2.446 ± 1.357E-02 |
| 20 | KT3P:125-150 | 99.051 ± 14.30 | 1.148 ± 6.200E-03 | 2.440 ± 3.057E-02 |
| 21 | KT3P:150-175 | 190,587.645 ± 8,824.40 | 1.130 ± 8.458E-04 | 2.423 ± 8.506E-03 |
| 22 | KT3P:175-200 | 386,199.417 ± 9,349.61 | 1.142 ± 6.601E-04 | 2.441 ± 4.261E-03 |
| 23 | KT3P:275-300 | 112,626.882 ± 1,179.15 | 1.169 ± 7.176E-04 | 2.437 ± 4.005E-03 |
| local Zn-Pb ore | | | | |
| 24 | local Zn-Pb ore | 111,759.092 ± 8,674.73 | 1.171 ± 9.120E-04 | 2.543 ± 5.706E-03 |
| Composite geological background samples | | | | |
| 25 | GB1 | 294.404 ± 12.18 | 1.167 ± 1.524E-04 | 2.495 ± 6.726E-03 |
| 26 | GB2 | 74.743 ± 1.08 | 1.158 ± 4.926E-04 | 2.451 ± 3.486E-03 |
| 27 | GB3 | 119.572± 1.13 | 1.153 ± 2.410E-05 | 2.450 ± 1.491E-03 |

**Table S5. Source apportionment based on Pb isotope ratios and Pb concentration**

| **Station** | **Source of Pb (%)** | | | | |
| --- | --- | --- | --- | --- | --- |
|  | **TS1** | **TS2** | **LS1** | **BS1** | **BS2** |
| **KC1** | 30 ± 1.42 | 0 | 0 | 70 ± 1.42 | 0 |
| **KC2** | 90 ± 0.00 | 10 ± 0.00 | 0 | 0 | 0 |
| **KC3** | 100 ± 4.10 | 0 | 0 | 0 | 0 |
| **KC4** | 90 ± 2.79 | 10 ± 2.79 | 0 | 0 | 0 |
| **KC4/1** | 50 ± 1.55 | 10 ± 1.55 | 0 | 40 ± 1.55 | 0 |
| **KC5** | 90 ± 0.24 | 10 ± 0.24 | 0 | 0 | 0 |


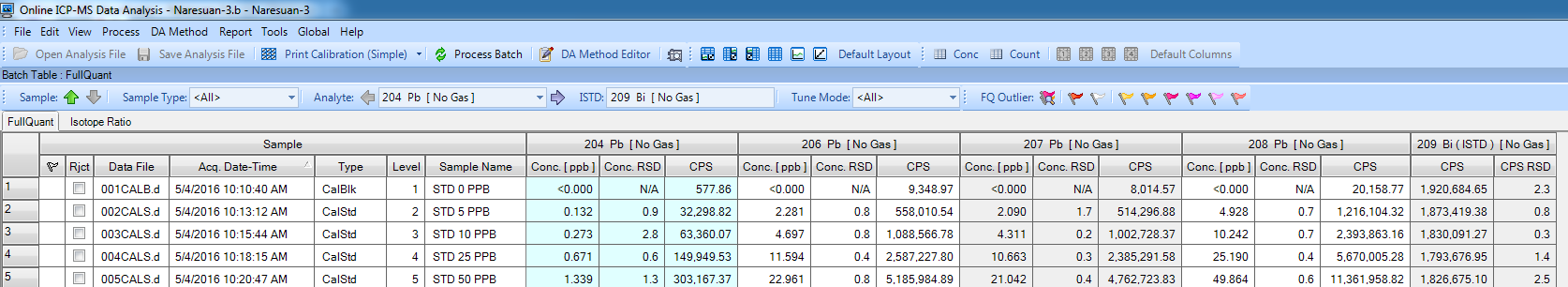


Figures S2. NIST SRM 981 Pb standard working data (^204^Pb ^206^Pb ^207^Pb and ^208^Pb) from Agilent ICP-MS MassHunter Software.


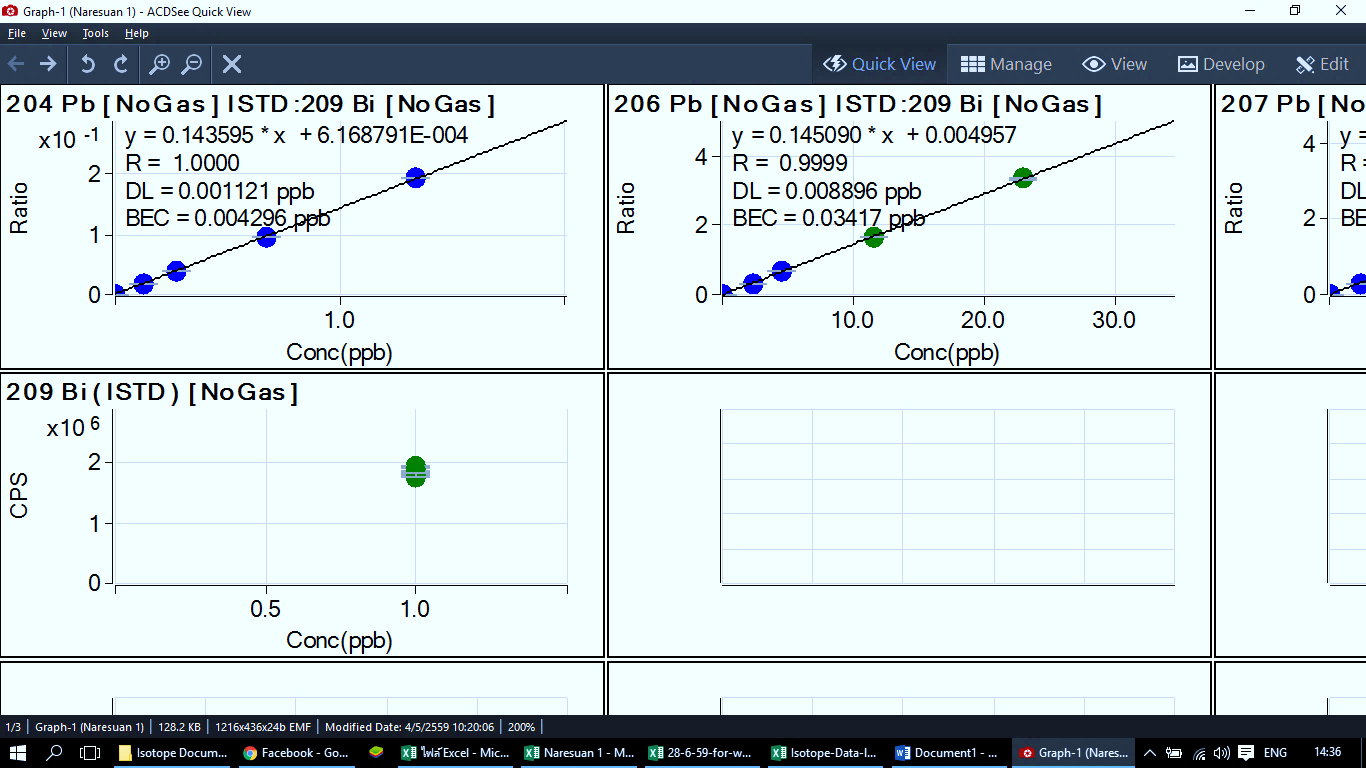


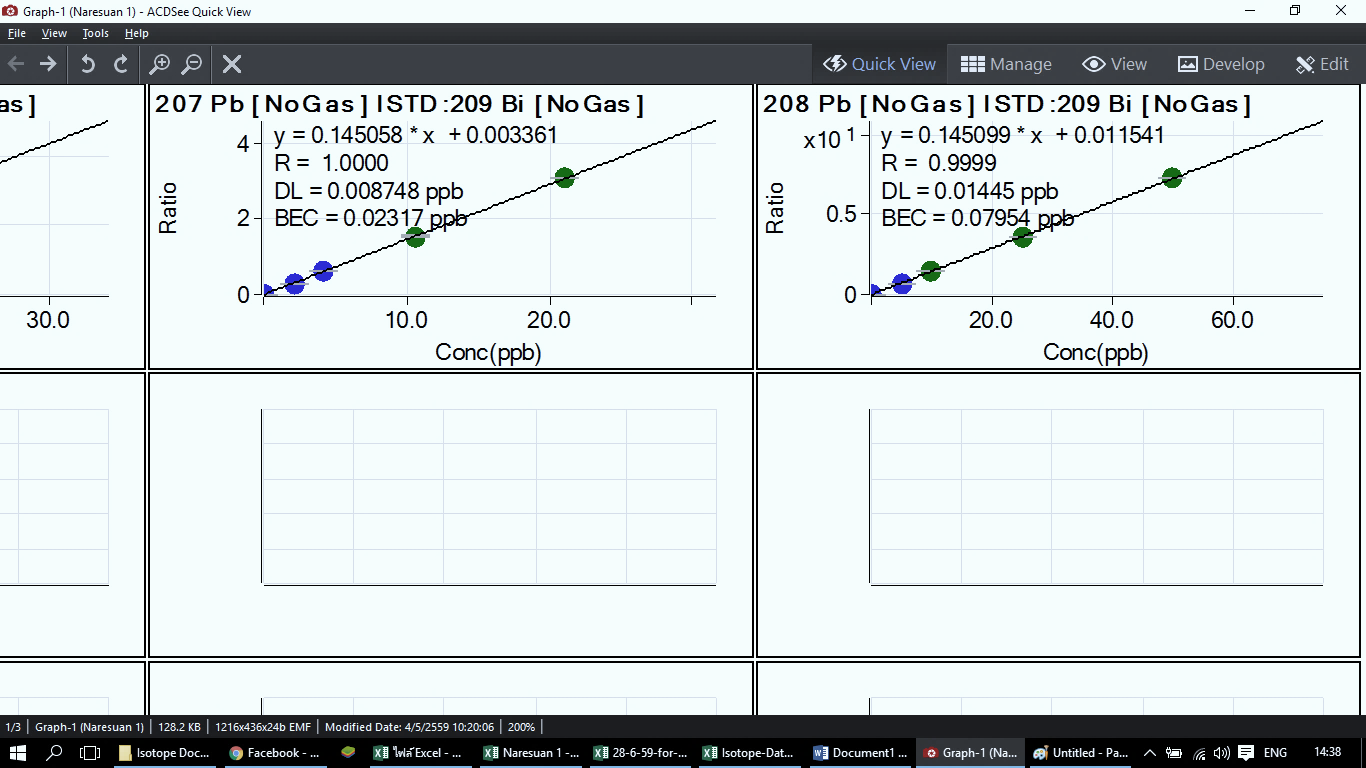


Figures S3. NIST SRM 981 Pb standard working curve (^204^Pb ^206^Pb ^207^Pb and ^208^Pb)


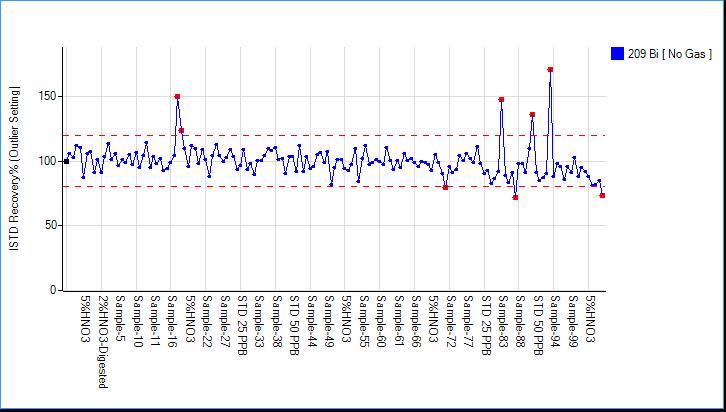


Figures S4. Internal standard recovery of ^209^Bi at 1 µg/L

**Reference**

Akande, S. O., & Zentilli, M. (1983). Genesis of the lead–zinc mineralization at Gays River, Nova Scotia, Canada. [https://dalspace.library.dal.ca/handle/10222/75100](about:blank)

Arribas, A., & Tosdal, R. M. (1994). Isotopic composition of Pb in ore deposits of the Betic Cordillera, Spain; origin and relationship to other European deposits. *Economic Geology, 89*(5), 20. doi:10.2113/gsecongeo.89.5.1074

Bjorlykke, A., Vokes, M., Birkeland, A., & Thorpe, I. (1993). Lead isotope systematics of strata-bound sulfide deposits in the Caledonides of Norway. *Economic Geology And The Bulletin Of The Society Of Economic Geologists, 88*(22), 20.

Brigo, L., Kostelka, L., Omenetto, P., Schneider, H.-J., Schroll, E., Schulz, O., & Štrucl, I. (1977). *Comparative Reflections on Four Alpine Pb-Zn Deposits*, Berlin, Heidelberg.

Cheng, H., & Hu, Y. (2010). Lead (Pb) isotopic fingerprinting and its applications in lead pollution studies in China: A review. *Environmental Pollution, 158*(5), 1134-1146. doi:[https://doi.org/10.1016/j.envpol.2009.12.028](about:blank)

Coron, C. R. (1981). *Facies Relations and Ore Genesis of the Newfoundland Zinc Mines Deposit, Daniel's Harbour, Western Newfoundland.* (Ph.D. thesis), University of Toronto, Toronto, ON.

Cumming, G. L., Kesler, S. E., & Krstic, D. (1979). Isotopic composition of lead in Mexican mineral deposits. *Economic Geology, 74*(6).

Cumming, G. L., Kyle, J. R., & Sangster, D. F. (1990). Pine Point; a case history of lead isotope homogeneity in a mississippi valley-type district. *Economic Geology, 85*(1), 133-144. doi:10.2113/gsecongeo.85.1.133

Cumming, G. L., & Richards, J. R. (1975). Ore lead isotope ratios in a continuously changing earth. *Earth and Planetary Science Letters, 28*(2), 155-171. doi:[https://doi.org/10.1016/0012-821X(75)90223-X](about:blank)

Deb, M., Thorpe, R. I., Cumming, G. L., & Wagner, P. A. (1989). Age, source and stratigraphic implications of Pb isotope data for conformable, sediment-hosted, base metal deposits in the Proterozoic Aravalli-Delhi orogenic belt, northwestern India. *Precambrian Research, 43*(1), 1-22. doi:[https://doi.org/10.1016/0301-9268(89)90002-8](about:blank)

Gemmel, B., Zantop, H., & Meinert, D. (1992). Genesis of the Aguilar zinc-lead-silver deposit, Argentina; contact metasomatic vs. sedimentary exhalative. *Economic Geology And The Bulletin Of The Society Of Economic Geologists, 87*(8), 27.

Godwin , C., Andrew , A., & Gabites , J. E. (1988). *Leadtable: A Galena Lead Isotope Data Base for the Canadian Cordillera, with a Guide to Its Use by Explorationists*: Mineral Resources Division, Geological Survey Branch.

Godwin, C., & Sinclair, A. (1982). Average lead isotope growth curves for shale-hosted zinc-lead deposits, Canadian Cordillera. *Economic Geology, 77*(3), 675-690. doi:10.2113/gsecongeo.77.3.675

Godwin, C., Sinclair, A., & Ryan, B. (1982). Lead isotope models for the genesis of carbonate-hosted Zn-Pb, shale-hosted Ba-Zn-Pb, and silver-rich deposits in the northern Canadian Cordillera. *Economic Geology, 77*(1), 82-94. doi:10.2113/gsecongeo.77.1.82

Gulson, B. L. (1984). Uranium-lead and lead-lead investigations of minerals from the Broken Hill lodes and mine sequence rocks. *Economic Geology, 79*, 476-490.

Gulson, B. L. (1985). Shale-hosted lead-zinc deposits in northern Australia. *Economic Geology, 80*(7), 12.

Gulson, B. L. (1986). *Lead isotope in mineral exploration*. Netherlands: Elsevier.

Heal, G. E. N. (1976). *The Wrigley–Lou and Polaris–Truro zinc deposits, N.W.T.* (M.Sc. thesis), University of Alberta, Edmonton, Alta.

Hou, B., & Zhao, D. (1993). Geology and Genesis of the Bajiazi Polymetallic Sulfide Deposits, Liaoning, China. *International Geology Review, October 1993*, 920-943. doi:10.1080/00206819309465565

Höy, T., & Godwin, C. I. (1988). Significance of a Cambrian date from galena lead-isotope data for the stratiform Cottonbelt deposit in the Monashee Complex, southeastern British Columbia *Canadian Journal of Earth Sciences, 25*(9), 8.

James, E. W., & Henry, C. D. (1993). Pb isotopes of ore deposits in Trans-Pecos Texas and northeastern Chihuahua, Mexico; basement, igneous, and sedimentary sources of metals. *Economic Geology, 88*(4), 13.

Koeppel, V. (1978). *Lead Isotope Studies of Stratiform Ore Deposits of the Namaqualand, NW Cape Province, South Africa, and Their Implications on the Age of the Bushmanland Sequence* Paper presented at the Proceedings of the Fifth Quadrennial IAGOD Symposium, Snowbird, Utah.

Koppel, V., & Schroll, E. (1983). Lead isotopes of Palaeozoic, strata-bound to stratiform galena bearing sulfide deposits of the Eastern Alps (Austria); implications for their geotectonic setting. *Schweizerische Mineralogische Und Petrographische Mitteilungen, 63*, 347-360.

Köppel, V., & Schroll, E. (1988). Pb-isotope evidence for the origin of lead in strata-bound Pb-Zn deposits in triassic carbonates of the Eastern and Southern Alps. *Mineralium Deposita, 23*(2), 96-103. doi:10.1007/BF00206657

Lang, D., & Zhang, X. (1987). Geological setting and genesis of the Jiashengpan PbZnS ore belt, Inner Mongolia. *Mineral Deposits, 6*(2), 39`53.

Lange, I. M., Nokleberg, W. J., Plahuta, J. T., Krouse, H. R., & Doe, B. R. (1985). Geologic setting, petrology, and geochemistry of stratiform sphalerite-galena-barite deposits, Red Dog Creek and Drenchwater Creek areas, northwestern Brooks Range, Alaska *Economic Geology And The Bulletin Of The Society Of Economic Geologists, 80*(7), 30.

LeCouteur, & Clifford, P. (1973). *A study of lead isotopes from mineral deposits in southeastern British Columbia and in the Anvil Range, Yukon Territory.* (Doctor of Philosophy - PhD), University of British Columbia, Vancouver : University of British Columbia Library. Retrieved from [https://open.library.ubc.ca/cIRcle/collections/ubctheses/831/items/1.0302659](about:blank)

Léon, D. (1998). Zinc-lead deposits of Belgium. *Ore Geology Reviews, 12*, 329-354. doi:10.1016/S0169-1368(98)00007-9

Li, N. (1998). *Depositional controls and genesis of the Jinding sandstone-hosted Zn–Pb deposit, Yunnan Province, southwest China.* (Ph.D. Thesis), University of Texas at Austin.

Macfarlane, A. W., Marcet, P., Lehuray, A. P., & Petersen, U. (1990). Lead Isotope Provinces of the Central Andes Inferred from Ores and Crustal Rocks. *Economic Geology And The Bulletin Of The Society Of Economic Geologists, 85*(8), 23.

McCracken, S. R., Geology, U. o. W. A. D. o., & Geophysics. (1997). *Stratigraphic, Diagenetic, and Structural Controls of the Admiral Bay Carbonate-hosted Zn-Pb-Ag Deposit, Canning Basin, Western Australia*: University of Western Australia.

Morganti, J. M. (1979). *The geology and ore deposits of the Howards Pass area, Yukon and Northwest Territories: the origin of basinal sedimentary stratiform sulphide deposits.* University of British Columbia, Vancouver, B.C.

Morrow, D., & Cumming, G. (1982). Interpretation of lead isotope data from zinclead mineralization in the northern part of the western Canadian Cordillera. *Canadian Journal of Earth Sciences, 19*(5), 11.

Puig, A. (1990). Lead isotopes in the Chilean ores. In L. Fontbote, G. C. Amstutz, M. Cardozo, E. Cedillo, & J. Frutos (Eds.), *Stratabound ore deposits in the Andes* (Vol. 8, pp. 749-758). Berlin: Springer-Verlag Berlin Heidelberg.

Rui, Z., Wang, L., & Li, N. (1991). *The Guanmenshan lead-zinc deposit: its basin brine ore-forming process and lead isotopic targeting*: Geological Publishing House.

Sangster, D. F., Savard, M. M., & Kontak, D. J. (1998). Sub-basin-specific Pb and Sr sources in Zn-Pb deposits of the Lower Windsor Group, Nova Scotia, Canada. *Economic Geology, 93*(6), 911-919. doi:10.2113/gsecongeo.93.6.911

Sangster, D. F., & Vaillancourt, P. D. (1990). *Geology of the Yava Sandstone - Lead Deposit, Cape Breton Island, Nova Scotia, Canada*.

Sato, K., & Sasaki, A. (1973). Lead Isotopes of the Black Ore ("Kuroko") Deposits from Japan *Economic Geology, 68*(4), 5.

Shanks, W. C., Woodruff, L. G., Jilson, G. A., Jennings, D. S., Modene, J. S., & Ryan, B. D. (1987). Sulfur and lead isotope studies of stratiform Zn-Pb-Ag deposits, Anvil Range, Yukon; basinal brine exhalation and anoxic bottom-water mixing. *Economic Geology, 82*(3), 600-634. doi:10.2113/gsecongeo.82.3.600

Song, X., Tan, H., & Sangster, D. F. (1996). Geochemical Characteristics of the Fankou Pb-Zn Deposit, Northern Guangdong, South China *Carbonate-Hosted Lead-Zinc Deposits: 75th Anniversary Volume* (Vol. 4, pp. 0): Society of Economic Geologists.

Tompkins, L., Pedone, V., Roche, M., & Groves, D. (1994). The Cadjebut Deposit as an example of Mississippi Valley-type mineralization on the Lennard Shelf, Western Australia; single episode or multiple events? *Economic Geology, 89*, 450-466. doi:10.2113/gsecongeo.89.3.450

Tornos, F., & Arias, D. (1993). Sulphur and lead isotope geochemistry of the Rubiales Zn-Pb ore deposit (NW Spain). *European Journal Mineralogy, 5, 763-773*.

Vaasjoki, M., & Gulson, B. L. (1986). Carbonate-hosted base metal deposits; lead isotope data bearing on their genesis and exploration. *Economic Geology, 81*(1), 16.

Vearncombe, J. R., Christensen, J. N., Dörling, S. L., McNaughton, N. J., & Reed, A. R. (1995). The Blendevale Ore-Body. In J.R. Veamcombe, S.L. Dörling, M.C. Dentith, A.W. Chisnall, J.N. Christensen, N.J. McNaughton, P.E. Playford, M.J. Rayner, & A.R Reed (Eds.), *Zinc-Lead Mineralization on the Southeast Lennard Shelf, Canning Basin, Western Australia* (Vol. 23, pp. 103-135).

Velasco, F., Pesquera, A., & Herrero, J. M. (1996). Lead isotope study of Zn-Pb ore deposits associated with the Basque-Cantabrian basin and Paleozoic basement, Northern Spain. *Mineralium Deposita, 31*(1), 84-92. doi:10.1007/BF00225398

Walker, R. N., Gulson, B., & Smith, J. (1983). The Coxco Deposit; a Proterozoic mississippi valley-type deposit in the McArthur River District, Northern Territory, Australia. *Economic Geology, 78*(2), 214-249. doi:10.2113/gsecongeo.78.2.214

Zartman, R. E., Pawlowska, J., & Rubinowski, Z. (1979). Lead isotopic composition of ore deposits from the Silesia-Cracow mining district. In E. A. Genezy & Badania (Eds.), *Research on the Genesis of Zinc-Lead Deposits of Upper Silesia, Poland* Prace Instytutu Geologicznego.
